# Developmental Disparities and Associated Factors Among Children Aged 36–59 Months in Four Pakistani Provinces

**DOI:** 10.3390/children13101314

**Published:** 2026-09-27

**Authors:** Syed Sikandar Shah, Ashfaq Ahmad Shah Bukhari, Jamil Ahmad, Aftab Alam, Junaid Asghar

**Affiliations:** 1 Department of Clinical Pharmacy and Pharmacology, RAK College of Pharmacy, RAK Medical and Health Sciences University, Ras Al Khaimah 11172, United Arab Emirates; aftab@rakmhsu.ac.ae; 2 Department of Physiology, RAK College of Medical Sciences, RAK Medical and Health Sciences University, Ras Al Khaimah 11172, United Arab Emirates; ashfaq.ahmad@rakmhsu.ac.ae; 3 Department of Psychiatric Nursing, RAK College of Nursing, RAK Medical and Health Sciences University, Ras Al Khaimah 11172, United Arab Emirates; jamil@rakmhsu.ac.ae; 4 Faculty of Pharmacy, Near East University, Near East Boulevard, Mersin 10, Nicosia 99138, Türkiye; muhammedjunaid.asghar@neu.edu.tr

**Keywords:** early childhood development index, early childhood education, nurturing care, stunting, socioeconomic inequality, multiple indicator cluster surveys

## Abstract

**Highlights:**

**What are the main findings?**

- Across 46,067 children, developmental attainment differed substantially across provinces and developmental domains, with no single provincial pattern characterizing all aspects of development.
- Socioeconomic gradients were evident in every province; ECE attendance was associated with higher developmental attainment, while stunting and functional difficulty were associated with lower attainment and adult-stimulation associations varied across settings.

**What are the implications of the main findings?**

- Population monitoring should combine overall ECDI attainment with domain-specific and socioeconomic profiles, as composite estimates alone may obscure important differences in developmental needs.
- Early-childhood planning should consider inequalities in early-learning access, nutritional conditions and inclusive developmental support, while longitudinal studies and program evaluations are needed to clarify the direction and effects of observed associations.

**Abstract:**

Background/Objectives: Early childhood developmental attainment varies across Pakistan, but provincial averages may obscure differences across developmental domains and socioeconomic groups. We examined developmental attainment and associated factors among children aged 36–59 months in four Pakistani provinces. Methods: We analyzed four provincially representative UNICEF-supported Multiple Indicator Cluster Surveys (MICS) conducted between 2017 and 2020. Development was assessed using the original Early Childhood Development Index (ECDI). Survey-design-based analyses estimated overall and domain-specific prevalence, adjusted prevalence ratios (aPRs), and wealth-related inequalities. Province-specific associations were summarized using random-effects meta-analysis. Results: Among 46,067 children, 59.4% met the overall ECDI criteria in Punjab, 54.6% in Khyber Pakhtunkhwa, 47.5% in Sindh, and 39.1% in Balochistan. This ordering persisted after age–sex standardization. Literacy–numeracy was the least frequently attained domain, ranging from 14.6% to 27.2%, whereas physical-domain attainment exceeded 90% in all four provinces. Early childhood education (ECE) attendance was associated with higher overall ECDI attainment (pooled aPR 1.29, 95% CI 1.22–1.37). Stunting and functional difficulty were associated with lower overall attainment, while associations with adult stimulation varied across provinces. Richest–poorest differences in overall ECDI prevalence ranged from 18.8 to 35.6 percentage points. Conclusions: Among children aged 36–59 months in four Pakistani provinces, developmental attainment differed substantially between provinces and across developmental domains, and these differences persisted after age–sex standardization. Socioeconomic inequalities were evident in every province. ECE attendance was associated with higher developmental attainment, but this association should be interpreted cautiously because the surveys were cross-sectional and may be affected by residual confounding and selection into ECE.

## 1. Introduction

Early childhood is a period of rapid development in language, learning, movement, and social interaction. The conditions in which children grow are closely related to these developmental processes and to later health, education, and participation in society [1,2,3,4]. In low- and middle-income countries, poverty, undernutrition, limited learning opportunities, and restricted access to services often occur together. Understanding how these circumstances are associated with early development is important for identifying population groups and settings in which additional support may be needed.

The Nurturing Care Framework brings together good health, adequate nutrition, responsive caregiving, opportunities for early learning, and safety [1]. These components connect children’s everyday experiences with the broader circumstances of their households and communities. Trials in Pakistan and other low-resource settings have demonstrated developmental benefits from responsive-stimulation and parenting interventions [5,6,7]. Population-based surveys complement such intervention evidence by describing the distribution of developmental attainment and the household and caregiving circumstances associated with it.

UNICEF’s original Early Childhood Development Index (ECDI) provides a standardized population-level measure for children aged 36–59 months in Multiple Indicator Cluster Surveys (MICS). Its 10 caregiver-reported items cover literacy–numeracy, physical, learning, and social-emotional development; children meeting the criteria in at least three of the four domains are classified as developmentally on track [8,9]. The instrument allows developmental attainment to be described using comparable questions and scoring rules across surveys. Examining the individual domains alongside the overall ECDI can provide additional information that may be obscured by the composite classification.

Pakistan’s pre-primary provision includes katchi classes and organized early childhood education (ECE), with substantial provincial differences in attendance documented in the provincial MICS reports [10,11,12,13]. The National Curriculum of Pakistan 2022–23 describes learning across language, number concepts, play, and social development, with expectations progressing between approximately ages 3–4 and 4–5 years. These include letter–sound recognition and counting, with progression toward simple word reading among older preschool children [14]. Because this curriculum was introduced after the MICSs analyzed here, it is used only to provide contemporary educational context and not as a retrospective standard for evaluating children surveyed during 2017–2020. The original ECDI literacy–numeracy thresholds should therefore be understood as fixed survey criteria rather than as skills that every child, particularly every 3-year-old, would necessarily be expected to have mastered.

Previous Pakistani analyses of MICS data have reported associations of developmental status with household wealth, parental education, nutritional status, functional difficulty, ECE attendance, and the home-learning environment [15,16]. However, less attention has been given to how these patterns differ across provincial populations and across individual developmental domains. The provincial surveys have independent sampling designs and were conducted in different periods, making it important to retain their individual survey structures rather than treating them as a single contemporaneous national sample. Examination of complementary measures of socioeconomic inequality can also distinguish the simple richest–poorest difference from the broader socioeconomic gradient across the population.

We analyzed four provincial MICS6 surveys to estimate overall and domain-specific developmental attainment, examine nutritional and nurturing-care associations, and characterize wealth-related inequalities. We preserved the sampling design of each survey, estimated province-specific associations, and summarized their consistency and heterogeneity across the four provincial survey populations. We also examined whether the association between stunting and overall ECDI varied according to reported adult stimulation. Exploratory analyses compared provincial developmental prevalence after standardization for age and sex.

## 2. Materials and Methods

### 2.1. Study Design and Data Sources

This cross-sectional study used the Punjab 2017–18, Sindh 2018–19, Khyber Pakhtunkhwa 2019, and Balochistan 2019–20 MICS6 surveys [10,11,12,13]. The respondent-level datasets were obtained through the UNICEF MICS survey portal following registration and approval [17]. The provincial statistical agencies conducted the surveys with UNICEF technical support. Each survey used stratified multistage sampling, with clusters selected within strata and households selected within clusters. Mothers or other eligible primary caregivers completed the under-five questionnaires.

We analyzed the four surveys separately using their original child sampling weights, strata, and primary sampling units (PSUs). We then summarized the provincial association estimates through random-effects meta-analysis. This approach retained the population represented by each survey; the four datasets were not treated as a single national sample. Reporting followed STROBE guidance for cross-sectional studies [18].

### 2.2. Study Population

The primary sample included children with a completed under-five interview and a recorded age of 3 or 4 completed years, consistent with the original MICS ECDI tabulations. We also examined eligibility based on calculated age of 36–59 months because a small number of records differed between the two definitions. Children could be represented by their mother or another eligible primary caregiver.

Prevalence analyses included all eligible children under the item-achievement rules described below. Fully adjusted models included children with complete data for the model covariates. We assessed the sensitivity of the results to missing data using missing-category models and inverse-probability-of-complete-case weighting (IPCW). Variable-level missingness is reported in Supplementary Table S12.

### 2.3. Outcome: Mics Early Childhood Development Index

We constructed the ECDI using the original MICS scoring rules [8,9]. Literacy–numeracy attainment required at least two of three abilities: identifying at least 10 letters, reading at least four simple words, and recognizing numbers 1–10. Physical-domain attainment required the ability to pick up a small object with two fingers or the absence of reported illness that sometimes prevents play. Learning attainment required following simple directions or completing a task independently. Social-emotional attainment required at least two of three behaviors: getting along with other children, not kicking, biting, or hitting, and not being easily distracted. Overall ECDI attainment required meeting the criteria in at least three domains.

Only responses confirming the specified ability or behavior contributed to item attainment. Unknown responses and nonresponse did not count as achieved items. We examined this scoring convention in a sensitivity analysis restricted to children with valid yes/no responses on all 10 items (Supplementary Table S15). Throughout the paper, developmental attainment refers to these survey criteria, rather than a clinical assessment.

We checked the reconstructed prevalence estimates against the corresponding overall and domain-specific estimates in Table TC.11.1 of each official provincial report (Supplementary Table S8).

### 2.4. Nutritional and Nurturing-Care Exposures

Stunting was the principal nutritional exposure and was defined as a valid height-for-age z score below −2 using the WHO Child Growth Standards [19]. Flagged, out-of-range, and missing anthropometric measurements were excluded from the stunting classification.

ECE exposure was current attendance in an organized early childhood education program. Adult stimulation indicated that a mother, father, or another adult had engaged the child in at least four of six activities during the preceding 3 days: reading or looking at picture books, telling stories, singing songs, going outside, playing, and naming, counting, or drawing. Maternal and paternal stimulation were also examined separately. Learning materials were represented by at least three children’s books and at least two types of playthings. Inadequate supervision indicated that the child had been left alone or with a child younger than 10 years for more than one hour on at least one day in the preceding week. Detailed coding and skip-response rules are provided in the Supplementary Methods.

### 2.5. Covariates

The fully adjusted models included age group, sex, urban–rural residence, functional difficulty, maternal/caretaker education, household wealth quintile, stunting, ECE attendance, adult stimulation, books, playthings, and supervision. Age groups corresponded to 3 and 4 completed years, presented as 36–47 and 48–59 months. Education was grouped as none/pre-primary, primary, middle, secondary, or higher. We used the harmonized MICS-derived education and functional-difficulty variables available in all four surveys.

The conceptual framework places provincial circumstances and household resources upstream of children’s nutritional and learning environments, while recognizing that prior child development may influence ECE enrollment and caregiver behavior (Supplementary Figure S1). The models estimate associations conditional on the other included variables. Because some variables may lie along pathways linking wealth or education with development, their coefficients do not represent separate total causal effects [20]. We also fitted models without functional difficulty to examine sensitivity to adjustment for a related developmental construct.

### 2.6. Data Analysis

Analyses used Python version 3.12.14 with NumPy, pandas, SciPy, and statsmodels. We estimated survey-weighted prevalence and 95% confidence intervals (CIs) using Taylor-series linearization, retaining each survey’s strata and PSUs. Singleton strata used centered adjustments. Technical implementation details are provided in the Supplementary Methods.

We fitted separate survey-weighted Poisson models with a log link and robust design-based standard errors for overall ECDI and each domain. This approach estimates adjusted prevalence ratios for common binary outcomes [21]. To examine the stimulation association, we also fitted sequential models adding demographic, socioeconomic, health, and other nurturing-care variables. Each sequence used the same complete-case sample, allowing changes with adjustment to be distinguished from changes in sample composition.

Provincial log aPRs were combined using restricted maximum likelihood random-effects models, with modified Hartung–Knapp–Sidik–Jonkman confidence intervals [22,23,24,25]. We assessed heterogeneity using Cochran’s Q, τ^2^, and I^2^ and calculated 95% prediction intervals. Provincial estimates and pooled summaries are presented together so that the average association can be considered alongside variation between surveys.

For provincial comparisons, we standardized prevalence to a common distribution of the four age-group-by-sex categories. The reference was the mean of the four survey-weighted provincial distributions, giving each province equal contribution. Standard errors treated this reference as fixed. We tested the six pairwise differences in standardized overall ECDI using independent-survey variances and Holm adjustment. Age- and sex-specific prevalence estimates retained survey design information (Supplementary Tables S9 and S13). These comparisons were exploratory.

We assessed wealth-related inequality using the richest–poorest prevalence difference and ratio, Slope Index of Inequality (SII), Relative Index of Inequality (RII), and Erreygers-corrected concentration index [26,27]. These measures describe absolute and relative differences and the distribution of attainment across the wealth ranking. The standard MICS wealth quintiles rank households within each province. As a sensitivity analysis, we constructed a common score from 14 identically coded household assets and applied pooled cut points after equalizing the total survey weight contributed by each province.

We tested stunting-by-stimulation interaction by adding a multiplicative term to each fully adjusted model and calculated standardized predicted prevalence for the four exposure combinations. Sensitivity analyses examined alternative age eligibility, missing covariate data, separate maternal and paternal stimulation, omission of functional difficulty, stricter ECE skip classification, and ECDI item nonresponse. ECE, stunting, functional difficulty, and household wealth were the principal theory-driven exposures; other associations and supplementary comparisons were exploratory. To describe multiplicity across the pooled analyses, we calculated Benjamini–Hochberg q values for one family of 45 tests, comprising nine predictors and five outcomes (Supplementary Table S11). We report two-sided *p* values and interpret estimates alongside their CIs, q values, and heterogeneity.

### 2.7. Ethical Considerations and Data Availability

The study used anonymized MICS data obtained through the authorized data-access process. The Faculty of Pharmacy, Gomal University, Pakistan, reviewed the study and determined that it was exempt from full ethical review (GU-634/959-Ph). The original surveys obtained informed consent and followed the ethical procedures described in their reports [10,11,12,13]. No additional participant contact or consent was required for this secondary analysis.

MICS respondent-level data remain available through UNICEF under its registration, approval, and data-use requirements. Analysis code and derived nonidentifiable outputs supporting the primary analyses and selected supplementary analyses are available from the corresponding author upon reasonable request, subject to the applicable data-use agreement.

## 3. Results

### 3.1. Participant Flow and Data Validation

Of 110,703 records in the four under-five files, 46,067 met the primary eligibility criteria: 15,973 in Punjab, 7892 in Sindh, 10,102 in Khyber Pakhtunkhwa, and 12,100 in Balochistan. Fully adjusted models included 40,945 children (88.9%), with provincial retention of 94.4%, 92.6%, 90.1%, and 78.1%, respectively (Table 1). In Balochistan, the largest proportions of missing covariate data concerned supervision (10.5%), stunting (7.1%), playthings (5.1%), and adult stimulation (2.0%). These exclusions overlapped (Supplementary Table S12).

Reconstructed overall and domain-specific prevalence estimates agreed with the official one-decimal survey tabulations to within approximately 0.05 percentage points (Supplementary Table S8).

### 3.2. Overall and Domain-Specific Developmental Status

The proportion meeting the overall ECDI criteria was 59.4% in Punjab (95% CI 58.6–60.3), 54.6% in Khyber Pakhtunkhwa (53.3–56.0), 47.5% in Sindh (45.9–49.0), and 39.1% in Balochistan (37.1–41.2) (Table 1; Figure 1).

Literacy–numeracy attainment was lowest among the four domains, ranging from 14.6% to 27.2%. Physical-domain attainment exceeded 90% in every province. Learning showed the largest provincial difference, ranging from 60.6% in Balochistan to 93.4% in Punjab. Social-emotional attainment ranged from 51.7% in Punjab to 60.8% in Balochistan, producing a different ordering from the overall index.

After age–sex standardization, overall prevalence was 59.5% in Punjab, 54.9% in Khyber Pakhtunkhwa, 47.3% in Sindh, and 39.0% in Balochistan. The Punjab–Balochistan difference was 20.5 percentage points (95% CI 18.3–22.7), and all six pairwise comparisons had Holm-adjusted *p* < 0.001 (Supplementary Table S13). Literacy–numeracy attainment was higher at 48–59 than at 36–47 months in every province: 39.1% versus 16.2% in Punjab, 28.5% versus 18.6% in Sindh, 31.5% versus 18.2% in Khyber Pakhtunkhwa, and 17.5% versus 11.6% in Balochistan. Sex differences were smaller and varied across provinces and domains (Supplementary Table S9).

Values are survey-weighted percentages (95% CIs), except *n*, which is unweighted. Eligible children had a completed under-five interview and a recorded age of 3 or 4 years. Model *n* denotes complete cases for the fully adjusted model. Overall ECDI requires attainment in at least three domains. Estimates incorporate the child sampling weight, strata, and PSUs of each provincial survey.

### 3.3. Distribution of Nutritional and Nurturing-Care Conditions

Stunting affected 33.7% of children in Punjab, 53.2% in Sindh, 41.3% in Khyber Pakhtunkhwa, and 50.5% in Balochistan (Table 2). ECE attendance was 34.6%, 10.3%, 7.6%, and 1.7%, respectively. Across provinces, only 1.9–4.3% of children had at least three children’s books. Adult engagement in four or more stimulation activities ranged from 28.0% to 49.3%, while paternal engagement at this threshold ranged from 2.8% to 10.8%. Inadequate supervision was most common in Balochistan (37.2%).

The sampled child populations also differed in residence and caregiver education. Urban residence accounted for 34.2% of children in Punjab, 44.7% in Sindh, 14.9% in Khyber Pakhtunkhwa, and 26.1% in Balochistan. The corresponding proportions whose mother or caretaker had no education or pre-primary education only were 45.1%, 67.0%, 69.6%, and 82.9% (Supplementary Table S12).

Values are survey-weighted percentages among children with observed data for each indicator. Stunting denotes a valid height-for-age z score below −2. Stimulation denotes at least four of six activities during the preceding 3 days. Inadequate supervision denotes being left alone or with a child younger than 10 years for more than one hour on at least one day in the preceding week. ECE, early childhood education.

### 3.4. Province-Specific Adjusted Associations

Functional difficulty was associated with lower overall ECDI attainment in all four adjusted provincial models, with aPRs ranging from 0.57 in Sindh to 0.84 in Punjab (Table 3). Stunting was inversely associated with attainment in Punjab, Sindh, and Balochistan; the Khyber Pakhtunkhwa estimate was closer to 1 and its CI included 1. ECE attendance was positively associated with attainment in Punjab, Sindh, and Khyber Pakhtunkhwa. The estimate in Balochistan was positive but less precise, in a population with low attendance.

Adult stimulation was positively associated with overall attainment in Punjab and Sindh, close to the null in Khyber Pakhtunkhwa, and inversely associated in Balochistan. In Balochistan, the estimate on a fixed sample was 0.922 before adjustment (95% CI 0.838–1.014) and 0.920 after full adjustment (0.850–0.995) (Supplementary Table S10). Books were positively associated with attainment in Punjab, Sindh, and Balochistan. Playthings showed positive associations in Sindh, Khyber Pakhtunkhwa, and Balochistan, but little association in Punjab.

Children in the richest quintile had higher adjusted ECDI prevalence than those in the poorest quintile in every province, with aPRs ranging from 1.13 in Khyber Pakhtunkhwa to 2.30 in Balochistan. Maternal/caretaker education was generally positively associated with attainment in Punjab and Khyber Pakhtunkhwa, with less consistent patterns in Sindh and Balochistan (Supplementary Table S1).

Values are aPRs (95% CIs) from separate survey-weighted modified Poisson models. All listed exposures were adjusted simultaneously for age, sex, residence, functional difficulty, maternal/caretaker education, wealth, stunting, ECE, adult stimulation, books, playthings, and supervision. Coefficients represent mutually adjusted associations. Inference uses unrounded values; a displayed CI limit of 1.00 may lie slightly above or below 1 before rounding.

### 3.5. Cross-Province Synthesis and Heterogeneity

The pooled aPR for ECE attendance and overall ECDI was 1.29 (95% CI 1.22–1.37), with I^2^ = 39.3% and a 95% prediction interval of 1.17–1.42 (Table 4; Figure 2). The pooled estimates were 0.92 for stunting (95% CI 0.85–0.98) and 0.73 for functional difficulty (0.55–0.96). Their prediction intervals included 1, reflecting greater variation across surveys.

Pooled associations with overall ECDI were less precise for stimulation, books, and playthings, and heterogeneity was high (I^2^ = 87.4–96.7%). For example, the books estimate was 1.25 (95% CI 0.99–1.57), with a prediction interval of 0.65–2.40. Older age was associated with higher attainment (pooled aPR 1.12, 95% CI 1.08–1.16). The pooled female-versus-male estimate was 1.02 (0.96–1.09).

Provincial log aPRs were pooled using restricted maximum likelihood with modified Hartung–Knapp–Sidik–Jonkman inference (four surveys). I^2^ describes observed between-survey heterogeneity. Prediction intervals describe the estimated range of associations in comparable settings and are uncertain with only four surveys. These pooled summaries are not nationally representative estimates. ECE, early childhood education.

### 3.6. Domain-Specific Associations

The largest ECE association was with literacy–numeracy attainment (pooled aPR 2.11, 95% CI 1.66–2.69; I^2^ = 89.5%) (Table 5). Books were also positively associated with this domain (1.40, 1.26–1.55; I^2^ = 0%), and stunting was inversely associated (0.79, 0.71–0.87). These associations remained below the 5% false-discovery threshold across 45 pooled tests: q = 0.0263 for ECE and books and q = 0.0374 for stunting. Adult stimulation had a positive estimate (1.49, 1.16–1.91; I^2^ = 81.0%), but its q value exceeded 0.05 (q = 0.0729) (Supplementary Table S11).

Functional difficulty had inverse pooled associations with physical, learning, and social-emotional attainment at the nominal 5% significance level. Playthings had a small positive association with physical attainment and an inverse association with social-emotional attainment. Of these additional domain associations, only the inverse playthings–social-emotional association met the 5% false-discovery threshold (q = 0.0499).

Cells show pooled aPRs (95% CIs), followed by I^2^. Exposure contrasts are specified in the column headings. Models use the adjustment set in Table 3. Domain analyses are exploratory; the complete *p* and q values for the 45 pooled tests appear in Supplementary Table S11.

### 3.7. Wealth-Related Developmental Inequality

Overall ECDI prevalence increased across wealth quintiles in every province (Table 6; Figure 3). Poorest-to-richest prevalence rose from 46.6% to 73.2% in Punjab, 36.4% to 71.2% in Sindh, 44.7% to 63.5% in Khyber Pakhtunkhwa, and 26.2% to 61.8% in Balochistan. The corresponding richest–poorest gaps were 26.6, 34.8, 18.8, and 35.6 percentage points.

The SII, RII, and Erreygers indices consistently showed higher overall attainment among wealthier households. The RII was 2.97 in Balochistan, 2.24 in Sindh, 1.71 in Punjab, and 1.51 in Khyber Pakhtunkhwa. Literacy–numeracy had the largest SII and RII in Punjab and Sindh, while Balochistan also showed marked inequalities in learning and social-emotional attainment (Supplementary Table S5).

Using the harmonized common-asset score retained the wealth gradient but reduced the top–bottom gaps to 20.6 percentage points in Punjab, 28.8 in Sindh, 16.9 in Khyber Pakhtunkhwa, and 20.9 in Balochistan (Supplementary Table S7). The direction of inequality was consistent across the two wealth measures, although the size and provincial ranking of the gaps changed.

Estimates are survey-weighted and unadjusted. The richest–poorest difference is expressed in percentage points (pp). SII describes the absolute difference across the full fractional wealth rank and is shown on the 0–1 proportion scale; multiplying by 100 gives percentage points. RII is the corresponding relative measure. A positive Erreygers index indicates concentration of attainment among wealthier households. Wealth quintiles represent relative position within each provincial survey.

### 3.8. Stunting and Adult-Stimulation Interaction

There was no statistically significant multiplicative stunting-by-stimulation interaction in Punjab (interaction PR 1.062, *p* = 0.065), Sindh (1.027, *p* = 0.608), Khyber Pakhtunkhwa (0.992, *p* = 0.866), or Balochistan (1.084, *p* = 0.232) (Supplementary Table S2). Across the four exposure combinations, standardized predicted prevalence ranged from 54.4% to 65.2% in Punjab, 42.5% to 52.7% in Sindh, 52.8% to 57.3% in Khyber Pakhtunkhwa, and 37.2% to 46.0% in Balochistan (Figure 4).

### 3.9. Maternal and Paternal Stimulation Sensitivity

Replacing the combined adult-stimulation indicator with separate maternal and paternal indicators produced heterogeneous provincial associations (Supplementary Table S6). Maternal stimulation was positively associated with overall attainment in Punjab and inversely associated in the other surveys. Paternal stimulation was positive in Punjab, close to the null in Khyber Pakhtunkhwa, and inverse in Sindh and Balochistan. Pooled estimates were 0.96 for maternal stimulation (95% CI 0.81–1.13; I^2^ = 92.3%) and 0.93 for paternal stimulation (0.69–1.25; I^2^ = 91.3%).

### 3.10. Sensitivity Analyses

Defining eligibility by calculated age of 36–59 months changed overall prevalence by less than 0.22 percentage points in each province (Supplementary Table S3). Missing-category models gave estimates close to the complete-case results. IPCW changed selected aPRs by no more than 3.0%, with the largest difference for functional difficulty in Balochistan (Supplementary Table S4).

Omitting functional difficulty while retaining the same complete cases produced similar ECE and stunting estimates. Stricter ECE skip classification excluded one additional complete case in Balochistan and none elsewhere (Supplementary Table S14). Restricting the sample to valid responses on all 10 ECDI items gave overall prevalence of 59.8% in Punjab, 48.2% in Sindh, 55.1% in Khyber Pakhtunkhwa, and 42.6% in Balochistan (Supplementary Table S15). Provincial ordering was unchanged, although the difference from the primary estimate was larger in Balochistan.

## 4. Discussion

### 4.1. Principal Findings

This study identified substantial provincial differences in developmental attainment among children aged 36–59 months. Punjab had the highest overall ECDI prevalence and Balochistan the lowest, and this ordering persisted after standardization for age and sex. However, the provincial pattern was not uniform across individual developmental domains. Balochistan, for example, had relatively low literacy–numeracy and learning attainment but the highest social-emotional point estimate. These findings show that the overall ECDI alone does not fully describe the developmental profile of a population and that domain-specific estimates provide important additional information.

ECE attendance was associated with higher overall developmental attainment, with the strongest pooled association observed for literacy–numeracy. Stunting and functional difficulty were associated with lower overall attainment, whereas associations with adult stimulation varied substantially across provinces. Wealth gradients were evident in all four provinces and remained present when an alternative common-asset measure was used. Together, these findings describe overlapping educational, nutritional, caregiving, and socioeconomic differences across the four provincial survey populations. The association with ECE should nevertheless be interpreted cautiously because selection into enrollment and residual confounding cannot be excluded in these cross-sectional data.

### 4.2. Relation to Pakistani and International Evidence

The findings extend previous Pakistani MICS analyses that have reported associations of developmental status with parental education, household wealth, nutrition, functional difficulty, ECE attendance, and the home-learning environment [15,16]. The present analysis adds province-specific estimates that retain the sampling design of each survey, distinguishes between developmental domains, and characterizes socioeconomic inequality using complementary measures. This is important because similar overall ECDI prevalence can arise from different domain profiles, while a similar richest–poorest difference can conceal variation across the remainder of the socioeconomic distribution.

The provincial ordering of overall ECDI attainment is consistent with the official MICS reports and previous Pakistani analyses [10,11,12,13,15,16]. Its persistence after age–sex standardization indicates that differences in the age and sex composition of the surveyed populations do not account for the observed provincial pattern. However, the contrasting social-emotional findings caution against describing any province as uniformly advantaged or disadvantaged across all aspects of development. Domain-specific profiles therefore provide a more informative basis for examining local learning environments, developmental needs, and service priorities.

Literacy–numeracy was the least frequently attained domain, but its interpretation requires consideration of both the skills assessed and the age range of the children. The original ECDI applies the same letter, word, and number thresholds throughout ages 36–59 months. In the present analysis, older children had substantially higher literacy–numeracy attainment in every province, consistent with developmental progression and increasing exposure to these skills. Pakistani curriculum guidance similarly describes advancing learning expectations across the preschool years [14]. Low attainment of the original ECDI literacy–numeracy thresholds should therefore not be interpreted as clinical developmental delay or as failure to meet an age-specific curriculum standard.

Studies from Ghana and Bangladesh have also reported associations of developmental attainment with ECE, household resources, caregiver education, and subnational context [28,29,30]. Comparisons of prevalence across studies nevertheless require attention to differences in the developmental instrument, age range, and population studied [31]. For example, recent work from Tanzania examined a different age range using a newer developmental measure [32]. ECDI2030 covers children aged 24–59 months, contains 20 items, and uses age-specific thresholds to generate an overall population indicator [33]. It is therefore distinct from the original four-domain ECDI used in the present analysis.

Sex differences were smaller than age differences and varied across provinces and developmental domains. This is consistent with the mixed pattern reported in the literature. One Pakistani analysis reported a modest male advantage [15], whereas a multicountry study found that observed differences more often favored girls, particularly in social-emotional development [34]. Reporting domain-specific estimates separately for boys and girls is therefore more informative than assuming a consistent developmental advantage for either sex.

### 4.3. Early Childhood Education and the Home-Learning Environment

The association between ECE attendance and literacy–numeracy is consistent with multicountry observational evidence showing stronger relationships between ECE attendance and this domain than with some other developmental outcomes [35]. Organized learning environments may provide repeated opportunities for exposure to letters, words, numbers, and structured learning activities. However, the magnitude of the association varied across provinces, suggesting that ECE attendance may represent different experiences, levels of access, and enrolled populations across settings.

ECE enrollment may be influenced by household resources, caregiver education, parental motivation, access to services, and children’s existing abilities or functional needs. These factors may contribute to the observed associations even after statistical adjustment. The surveys also contain limited comparable information on program quality, duration of attendance, language of instruction, frequency of participation, or age at entry. ECE attendance was particularly uncommon in Balochistan, reducing precision for that province. The findings therefore support further examination of ECE access and quality, while longitudinal studies and intervention evaluations are needed to estimate the developmental effects of enrollment.

Access to children’s books was uncommon in all four provinces, yet book availability showed a positive pooled association with literacy–numeracy and little observed heterogeneity for this domain. Book ownership alone does not indicate how frequently or effectively books are used with children, but it may reflect the availability of learning materials within the home. A meta-analysis of randomized book-sharing interventions found improvements in child language and caregiver book-sharing competence [36]. Taken together, the observational and intervention evidence supports attention not only to the availability of appropriate materials but also to caregiver support for their use.

Adult stimulation showed a less consistent pattern. Its pooled association with literacy–numeracy was positive but did not meet the 5% false-discovery threshold, and its association with overall ECDI varied markedly across provinces. The MICS stimulation indicator records whether specified activities occurred during the preceding 3 days [9,37], but it does not measure their quality, duration, responsiveness, or developmental appropriateness. It therefore differs substantially from the structured responsive-stimulation interventions evaluated in randomized trials [5,6,7].

The inverse association between adult stimulation and overall ECDI in Balochistan was already present before adjustment on the same analytical sample, indicating that it was not created by a change in sample composition or by reversal from a positive unadjusted association. One possible explanation is that caregivers may increase developmental activities in response to perceived child difficulties. Unmeasured adversity, differences in reporting, or residual confounding may also contribute. In addition, adjustment for correlated household and caregiving variables can alter coefficients through overadjustment or collider bias [20]. The unadjusted confidence interval included 1, and the fully adjusted estimate remained close to 1. This finding should therefore be interpreted cautiously and does not indicate that stimulation is harmful.

### 4.4. Nutrition and Development

The inverse association between stunting and developmental attainment was strongest for literacy–numeracy. A meta-analysis of MICS data from 15 low- and middle-income countries similarly reported variation in stunting–development associations across developmental domains and settings [38]. In the present analysis, the association with overall ECDI was weaker in Khyber Pakhtunkhwa, and the pooled prediction interval included 1. Stunting therefore identified developmental disadvantage at the population level without demonstrating a uniform association across all provinces and developmental outcomes.

The distinction between nutritional status and developmental progress is important. A systematic review examining linear growth beyond age 2 reported small or inconsistent relationships with later neurodevelopment [39]. Stunting may reflect accumulated nutritional, infectious, environmental, and socioeconomic adversity, several components of which may also influence child development [2,40,41]. Its association with ECDI should therefore be interpreted within this broader context rather than attributed to a single biological or nutritional pathway.

More than 90% of children met the physical-domain criteria in every province. This high level of attainment may limit the ability of the original ECDI physical domain to distinguish between children with different nutritional or motor-development profiles. The contrast between the high physical-domain prevalence and substantially lower literacy–numeracy or learning attainment further illustrates why the four domain percentages should not be interpreted as interchangeable measures of developmental difficulty.

The interaction analyses did not provide clear evidence that the association between stunting and ECDI differed according to the dichotomized adult-stimulation measure. These analyses concerned a brief caregiver-reported activity indicator and developmental status measured at the same survey occasion, whereas intervention trials evaluate structured programs delivered over time [5,6,7]. The present findings therefore do not determine whether sustained, high-quality responsive caregiving can modify the developmental consequences of nutritional adversity. Integrated attention to nutrition and responsive caregiving remain supported by the broader intervention evidence.

### 4.5. Socioeconomic Inequality and Provincial Context

The consistent wealth gradient across several inequality measures indicates that developmental attainment varied across the broader socioeconomic distribution rather than only between the poorest and richest households. Similar socioeconomic patterns have been reported in Pakistan, Ghana, and Bangladesh [15,16,28,29,30]. Multicountry evidence has also documented inequalities in home- and center-based learning opportunities [42]. Household resources may influence nutrition, caregiver time, access to learning materials, transport, and use of educational and health services, providing several possible pathways through which socioeconomic circumstances may be related to early development.

Provincial context helps situate these inequalities. UNDP’s assessment based on 2014–15 data placed Punjab above Sindh and Khyber Pakhtunkhwa on Pakistan’s human-development measure, with Balochistan lowest, while also documenting substantial variation between districts [43]. The present surveys similarly showed provincial differences in urban residence, caregiver education, stunting, and ECE attendance. These measured characteristics provide more direct context for the surveyed child populations than broad descriptions of provinces as primarily industrial or agricultural. At the same time, provincial averages inevitably conceal substantial variation within urban areas, rural areas, and districts.

The association between household wealth and developmental attainment remained after adjustment for measured nutritional and learning-related factors. This indicates that these measured variables did not account for the full socioeconomic pattern, although the models cannot identify the contribution of individual pathways or establish causal mediation. More detailed information on service access, household constraints, caregiver resources, and the quality of children’s everyday experiences would be required to investigate these mechanisms.

The alternative common-asset analysis supported the presence of a socioeconomic gradient while qualifying comparisons of its magnitude across provinces. Standard MICS wealth quintiles describe relative socioeconomic position within each provincial asset distribution. Applying a common asset score and common cut points reduced several richest–poorest differences, particularly in Balochistan, and changed the provincial ordering of the gaps. Thus, the existence of socioeconomic inequality was more consistent than the ranking of provinces according to its magnitude.

Associations also varied substantially between provincial surveys. For overall ECDI, adult stimulation, books, and playthings had I^2^ values above 87%, making the province-specific estimates particularly important for interpretation. With only four surveys, estimates of between-survey heterogeneity and prediction intervals remain uncertain despite the use of modified Hartung–Knapp inference [24,25]. The pooled estimates should therefore be interpreted as summaries of consistency and variation across the four surveys rather than as nationally representative effects.

### 4.6. Functional Difficulty and Inclusive Development

Children with reported functional difficulties had lower overall ECDI attainment in each province, consistent with findings from other South Asian MICS analyses [44]. Interpretation requires recognition that functional-difficulty measures and the original ECDI assess partly related areas of child functioning. Communication, learning, behavior, and motor difficulties may overlap conceptually with some ECDI items, and both measures are reported by the caregiver. Shared reporting processes may therefore contribute to the observed association. Omitting functional difficulty from the adjusted models produced little change in the main ECE and stunting estimates, but this sensitivity analysis does not eliminate the conceptual overlap between the two measures.

These findings reinforce the importance of including children with functional difficulties in developmental monitoring and service planning. Evidence from low- and middle-income countries supports inclusive, caregiver-involved early-intervention approaches while also highlighting limitations in service coordination, accessibility, and formal evaluation [45]. Developmental services should therefore respond to individual needs rather than relying on the overall ECDI classification alone.

### 4.7. Strengths and Limitations

This study has several strengths. It draws on large, population-based provincial samples and uses a common developmental instrument across all four surveys. Preservation of each survey’s sampling design, comparison of reconstructed estimates with official MICS tabulations, and presentation of province-specific estimates alongside pooled summaries improve the transparency of the analysis. Examination of individual developmental domains and complementary measures of socioeconomic inequality provides information beyond overall ECDI prevalence. Multiple sensitivity analyses also assessed uncertainty related to age eligibility, missing data, exposure coding, ECDI item responses, and model specification.

Several limitations should also be considered. First, cross-sectional design prevents conclusions about temporal direction or causality. Prior child development may influence ECE enrollment and caregiver behavior, and statistical adjustment cannot eliminate confounding by unmeasured characteristics such as prematurity, caregiver mental health, food insecurity, interaction quality, or local service availability. Some included covariates may also lie on pathways between other exposures and developmental outcomes. Second, many measures were caregiver reported and may therefore be affected by recall, social desirability, and differences in interpretation. The original ECDI is a population-monitoring instrument with fixed thresholds and limited coverage of each domain rather than a clinical diagnostic assessment [8,9,31,33].

The four provincial surveys were conducted in different periods, meaning that geographic differences cannot be fully separated from differences in survey year. School calendars, seasonal illness, fieldwork conditions, and changes in services may also have influenced the measurements. Standardized survey questions and age–sex standardization improve comparability, but the findings should be interpreted as estimates for four province–period populations rather than as a contemporaneous national comparison.

Missing covariate data was more common in Balochistan. The similarity between complete-case and weighting-based estimates is reassuring with respect to measured variables but cannot exclude bias arising from unmeasured determinants of missingness. ECDI item nonresponse also affected prevalence estimates: restricting the analysis to children with valid responses on all 10 items increased the Balochistan estimate from 39.1% to 42.6%. Because this restriction changes the analytical sample, neither estimate can be assumed to eliminate nonresponse bias. Finally, only four surveys contributed to the meta-analyses, limiting the precision of heterogeneity and prediction-interval estimates. The number of associations examined also requires attention to the reported q values and to the exploratory nature of several supplementary analyses.

### 4.8. Policy and Research Implications

Provincial monitoring of early childhood development should consider overall developmental attainment together with domain-specific and socioeconomic information. The contrasting developmental profiles and persistent wealth gradients identify areas in which more detailed assessment of early-learning access, nutritional conditions, household resources, and inclusive developmental services may be useful. Program decisions can draw on such population-level distributional evidence together with evidence from trials of responsive caregiving, nutrition, and book-sharing interventions [5,6,7,36]. Attention to implementation quality is particularly important when expanding services to underserved populations and to children with functional difficulties [45].

Future surveys using ECDI2030 can support updated population monitoring, while complementary validated developmental measures will remain necessary for more detailed domain-specific assessment [33]. Repeated provincial surveys linked with information on local service availability and quality could help distinguish changes over time from geographic differences. Longitudinal studies and well-designed program evaluations are also needed to clarify selection into ECE, the direction of associations between caregiving and child development, and whether improvements in access and service quality reduce developmental inequalities.

## 5. Conclusions

Developmental attainment differed substantially among the four provincial survey populations, and these differences persisted after age–sex standardization. However, no single provincial ordering was observed across all developmental domains. Socioeconomic gradients were evident across alternative wealth measures, although their magnitude varied according to how household socioeconomic position was defined. These findings support monitoring approaches that consider overall ECDI attainment together with domain-specific patterns and household circumstances.

ECE attendance was associated with higher developmental attainment, whereas associations with adult stimulation varied across settings. These findings highlight areas for further assessment and service planning related to early learning, nutrition, and inclusive developmental support. Because the data is cross-sectional, the observed associations should not be interpreted as intervention effects.

## Figures and Tables

**Figure 1:**
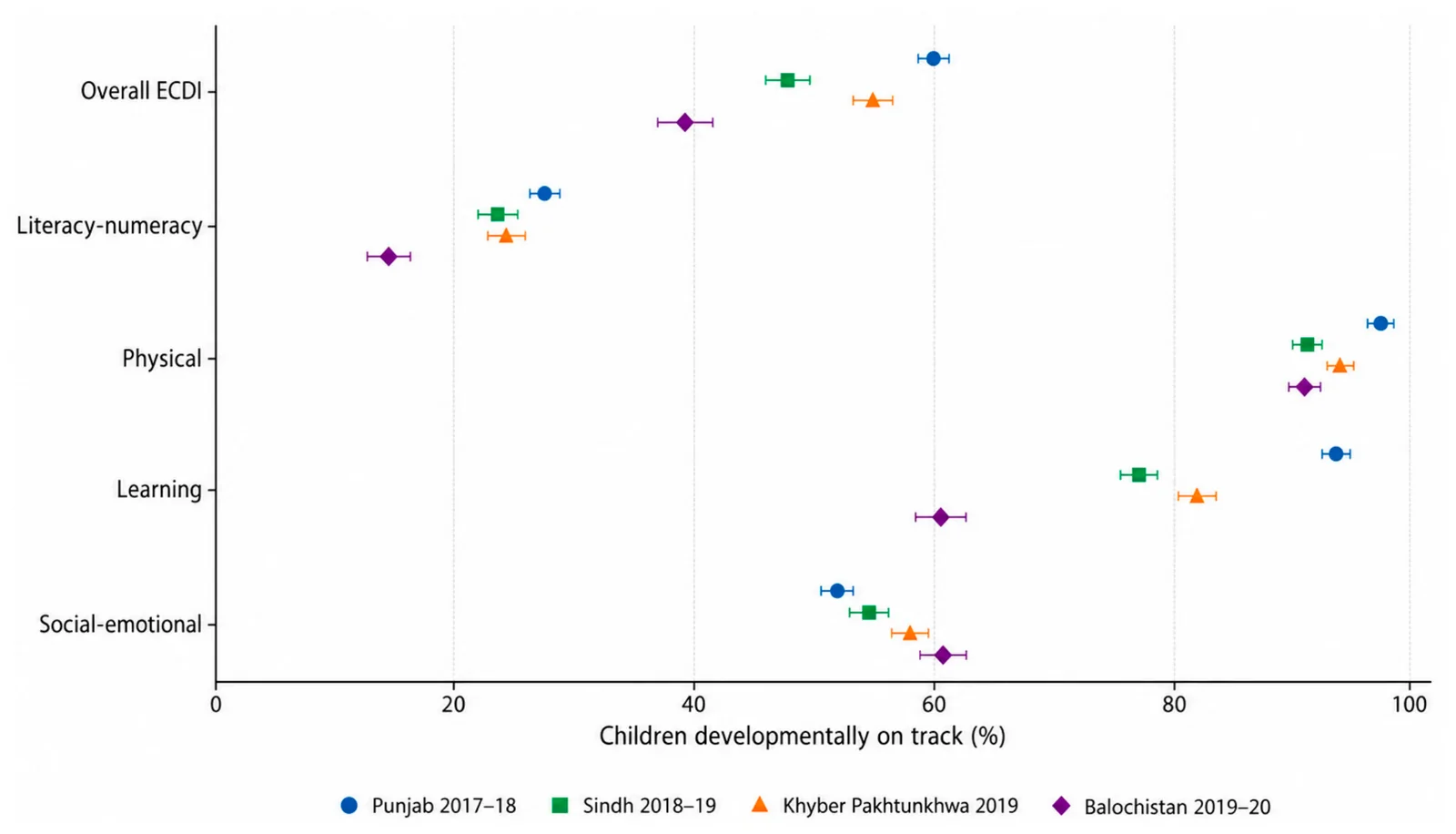
Overall and domain-specific ECDI attainment in four provincial MICS6 surveys. Points show survey-weighted percentages; horizontal bars show 95% CIs accounting for each survey’s weights, strata, and PSUs. Overall ECDI requires attainment in at least three domains. Learning attainment differs by 32.8 percentage points between Punjab and Balochistan; the error bars describe precision within each survey.

**Figure 2:**
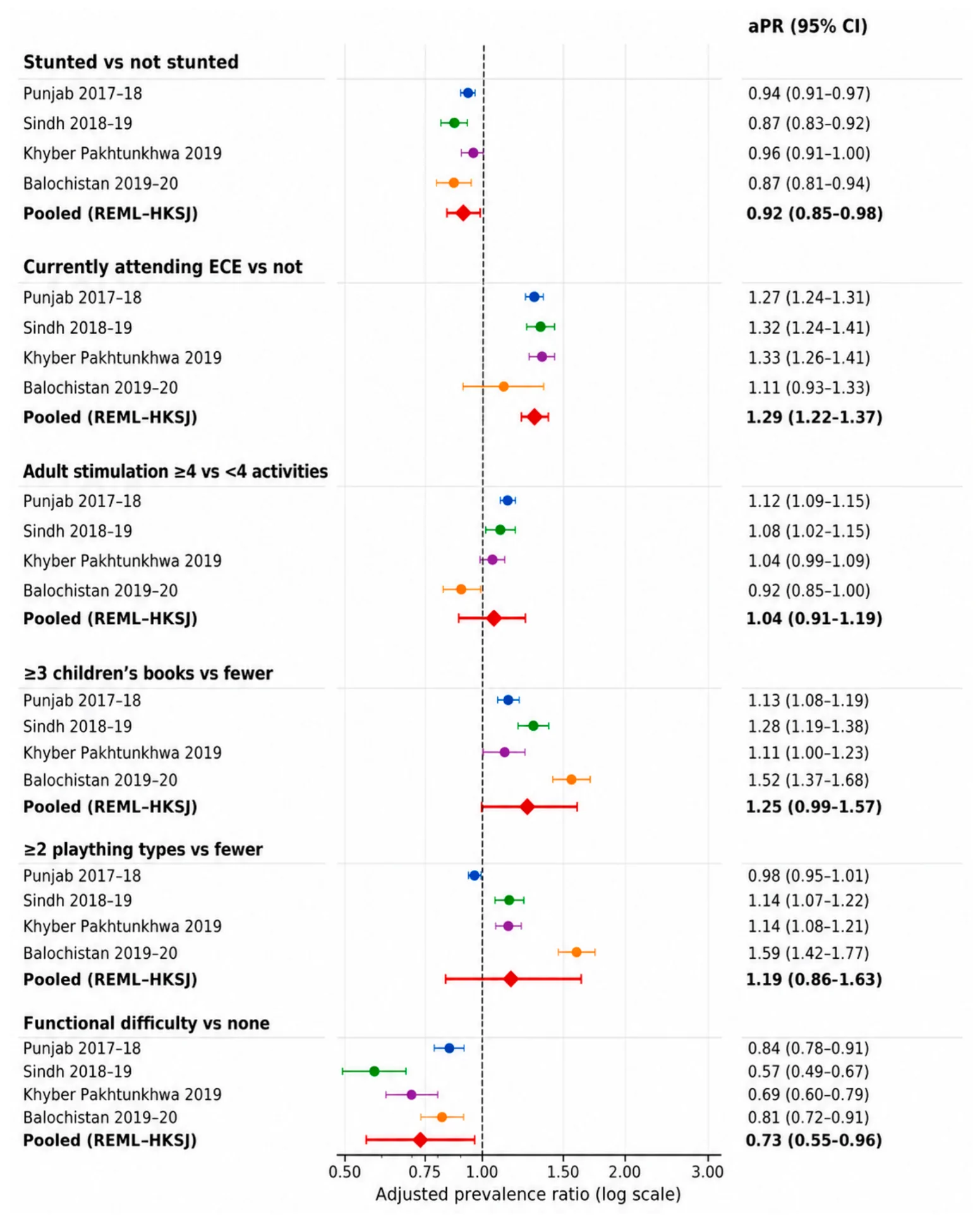
Selected provincial and pooled adjusted associations with overall ECDI. Circular markers show provincial aPRs; diamonds show random-effects summaries. Horizontal bars indicate 95% CIs, and the dashed line marks aPR = 1. Models include the covariates listed in Table 3. Heterogeneity and prediction intervals are reported in Table 4.

**Figure 3:**
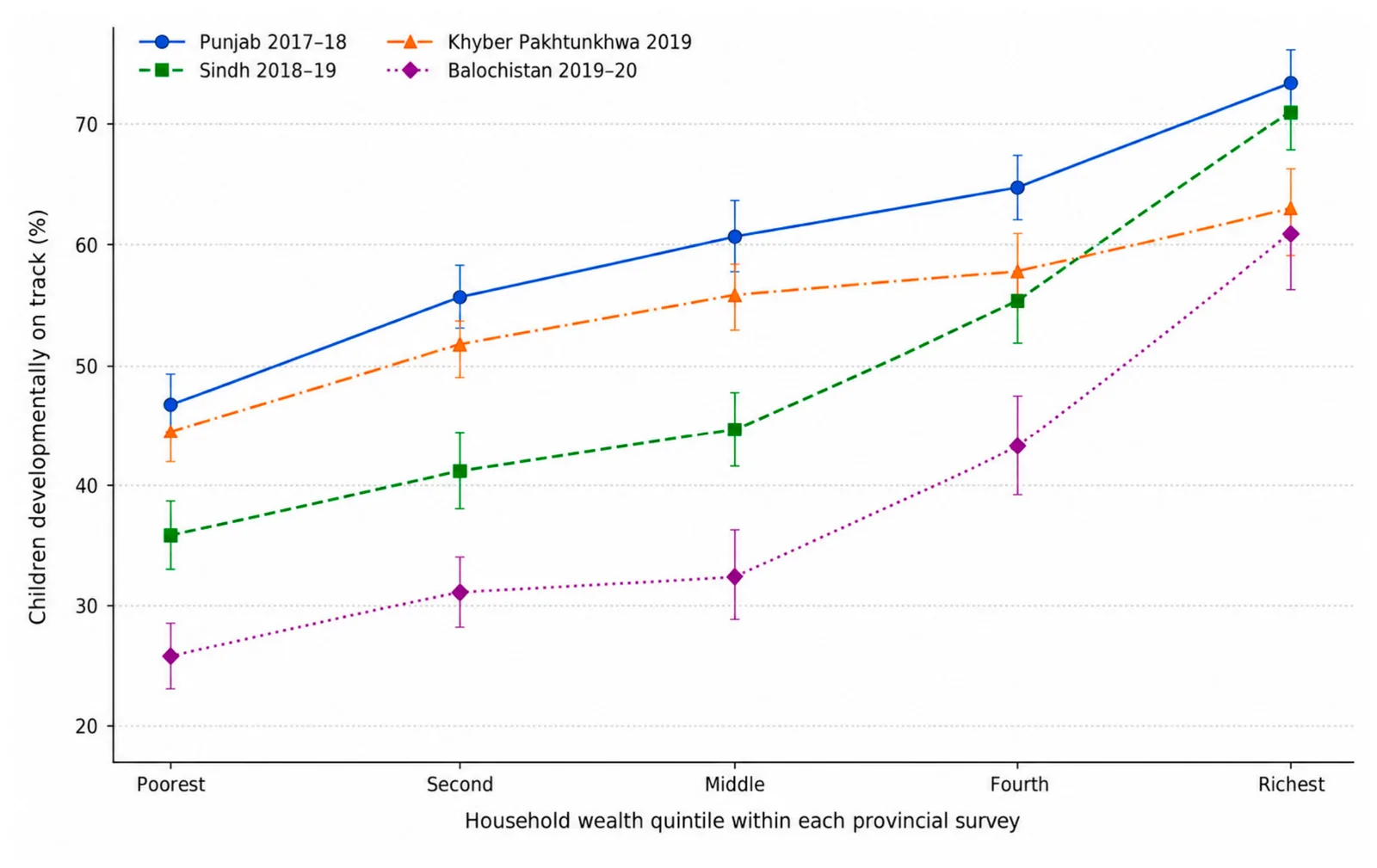
Overall ECDI attainment across provincial household wealth quintiles. Points show survey-weighted percentages and vertical bars show 95% CIs. Connecting lines illustrate the ordered wealth gradient. Quintiles rank households within each province; results using a common-asset definition are provided in Supplementary Table S7.

**Figure 4:**
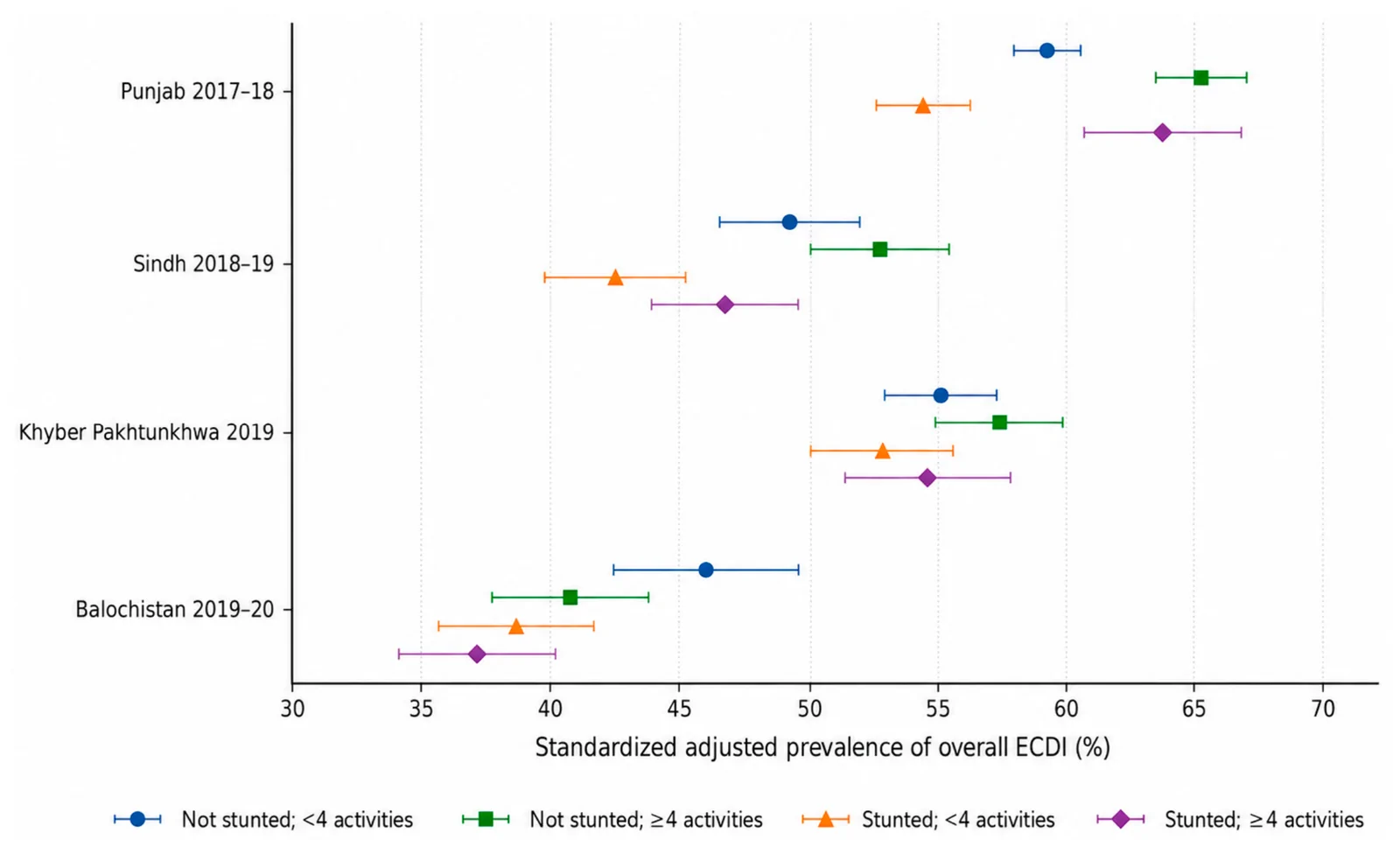
Standardized adjusted prevalence of overall ECDI by stunting and adult stimulation. Points show predicted percentages from the fully adjusted provincial models; horizontal bars show 95% CIs. Stimulation denotes at least four of six activities in the preceding 3 days. Multiplicative interaction tests are reported in Supplementary Table S2.

**Table 1:** Analytical sample sizes and survey-weighted prevalence of overall and domain-specific Early Childhood Development Index outcomes among children aged 36–59 months.

Provincial Survey	Eligible *n*	Model *n*	Overall ECDI	Literacy-Numeracy	Physical	Learning	Social-Emotional
Punjab 2017–18	15,973	15,082	59.4 (58.6–60.3)	27.2 (26.4–28.0)	97.2 (97.0–97.5)	93.4 (92.9–93.8)	51.7 (50.8–52.6)
Sindh 2018–19	7892	7309	47.5 (45.9–49.0)	23.6 (22.3–24.9)	91.1 (90.3–92.0)	77.0 (75.9–78.2)	54.4 (53.0–55.9)
Khyber Pakhtunkhwa 2019	10,102	9102	54.6 (53.3–56.0)	24.4 (23.1–25.7)	93.8 (93.2–94.4)	81.9 (80.8–83.0)	57.9 (56.6–59.2)
Balochistan 2019–20	12,100	9452	39.1 (37.1–41.2)	14.6 (13.4–15.9)	90.8 (90.1–91.6)	60.6 (58.8–62.4)	60.8 (59.0–62.5)

**Table 2:** Survey-weighted distribution of nutritional, early-learning, caregiving, supervision, and functional-difficulty indicators.

Indicator	Punjab 2017–18	Sindh 2018–19	Khyber Pakhtunkhwa 2019	Balochistan 2019–20
Stunted	33.7	53.2	41.3	50.5
Currently attending ECE	34.6	10.3	7.6	1.7
Adult stimulation (≥4 activities)	28.0	45.8	35.2	49.3
Maternal stimulation (≥4 activities)	11.2	16.8	7.5	30.1
Paternal stimulation (≥4 activities)	3.0	8.3	2.8	10.8
At least 3 children’s books	4.3	4.3	1.9	4.0
At least 2 plaything types	60.6	72.3	67.9	65.5
Inadequate supervision	11.9	21.7	21.5	37.2
Functional difficulty	6.0	6.0	5.6	12.3

**Table 3:** Province-specific adjusted associations of selected child, nutritional, nurturing-care, education, and wealth factors with overall Early Childhood Development Index status.

Predictor	Punjab 2017–18	Sindh 2018–19	Khyber Pakhtunkhwa 2019	Balochistan 2019–20
Functional difficulty	0.84 (0.78–0.91)	0.57 (0.49–0.67)	0.69 (0.60–0.79)	0.81 (0.72–0.91)
Stunting	0.94 (0.91–0.97)	0.87 (0.83–0.92)	0.96 (0.91–1.00)	0.87 (0.81–0.94)
Current ECE attendance	1.27 (1.24–1.31)	1.32 (1.24–1.41)	1.33 (1.26–1.41)	1.11 (0.93–1.33)
Adult stimulation ≥ 4 activities	1.12 (1.09–1.15)	1.08 (1.02–1.15)	1.04 (0.99–1.09)	0.92 (0.85–1.00)
At least 3 children’s books	1.13 (1.08–1.19)	1.28 (1.19–1.38)	1.11 (1.00–1.23)	1.52 (1.37–1.68)
At least 2 plaything types	0.98 (0.95–1.01)	1.14 (1.07–1.22)	1.14 (1.08–1.21)	1.59 (1.42–1.77)
Inadequate supervision	1.04 (1.00–1.08)	0.97 (0.91–1.04)	1.02 (0.97–1.08)	0.98 (0.90–1.06)
Higher vs. no/pre-primary maternal education	1.10 (1.05–1.16)	1.08 (0.98–1.19)	1.19 (1.10–1.29)	0.79 (0.66–0.94)
Richest vs. poorest wealth quintile	1.24 (1.16–1.32)	1.40 (1.23–1.59)	1.13 (1.03–1.25)	2.30 (1.98–2.66)

**Table 4:** Random-effects synthesis of province-specific adjusted associations with overall Early Childhood Development Index status.

Predictor	Pooled aPR (95% CI)	*p*	I^2^ (%)	95% Prediction Interval
Functional difficulty vs. none	0.73 (0.55–0.96)	0.036	86.5	0.33–1.60
Stunted vs. not stunted	0.92 (0.85–0.98)	0.030	63.8	0.76–1.10
Currently attending ECE vs. not	1.29 (1.22–1.37)	<0.001	39.3	1.17–1.42
Adult stimulation ≥ 4 vs. <4 activities	1.04 (0.91–1.19)	0.391	87.4	0.72–1.52
≥3 children’s books vs. fewer	1.25 (0.99–1.57)	0.054	90.4	0.65–2.40
≥2 plaything types vs. fewer	1.19 (0.86–1.63)	0.186	96.7	0.46–3.06
Inadequate supervision vs. none	1.01 (0.96–1.07)	0.491	23.8	0.91–1.13
Age 48–59 vs. 36–47 months	1.12 (1.08–1.16)	0.002	21.3	1.06–1.18
Female vs. male	1.02 (0.96–1.09)	0.328	72.6	0.87–1.21

**Table 5:** Domain-specific random-effects pooled associations for selected nutritional and nurturing-care exposures. Panel (**A**), nutritional status and early learning. Panel (**B**), learning materials and functional difficulty.

**(A)**			
**Outcome**	**Stunted vs. Not Stunted**	**Current ECE Attendance vs. None**	**Adult Stimulation ≥ 4 vs. <4 Activities**
Overall ECDI	0.92 (0.85–0.98) I^2^ = 63.8%	1.29 (1.22–1.37) I^2^ = 39.3%	1.04 (0.91–1.19) I^2^ = 87.4%
Literacy–numeracy	0.79 (0.71–0.87) I^2^ = 31.6%	2.11 (1.66–2.69) I^2^ = 89.5%	1.49 (1.16–1.91) I^2^ = 81.0%
Physical	1.00 (0.98–1.02) I^2^ = 69.2%	1.03 (1.00–1.05) I^2^ = 73.3%	1.00 (0.97–1.03) I^2^ = 80.4%
Learning	0.97 (0.91–1.03) I^2^ = 84.6%	1.06 (0.99–1.14) I^2^ = 88.1%	0.96 (0.84–1.09) I^2^ = 94.4%
Social–emotional	0.98 (0.94–1.02) I^2^ = 9.9%	0.96 (0.80–1.15) I^2^ = 68.2%	0.98 (0.92–1.05) I^2^ = 66.4%
**(B)**			
**Outcome**	**≥3 Children’s Books vs. Fewer**	**≥2 Plaything Types vs. Fewer**	**Functional Difficulty vs. None**
Overall ECDI	1.25 (0.99–1.57) I^2^ = 90.4%	1.19 (0.86–1.63) I^2^ = 96.7%	0.73 (0.55–0.96) I^2^ = 86.5%
Literacy–numeracy	1.40 (1.26–1.55) I^2^ = 0.0%	1.28 (0.91–1.80) I^2^ = 94.3%	0.91 (0.75–1.10) I^2^ = 45.1%
Physical	1.02 (0.97–1.06) I^2^ = 66.5%	1.01 (1.00–1.02) I^2^ = 0.0%	0.95 (0.91–0.98) I^2^ = 45.1%
Learning	1.08 (0.90–1.31) I^2^ = 95.7%	1.22 (0.92–1.61) I^2^ = 98.6%	0.84 (0.73–0.96) I^2^ = 84.2%
Social–emotional	1.11 (0.95–1.29) I^2^ = 70.2%	0.88 (0.82–0.94) I^2^ = 68.1%	0.92 (0.86–0.98) I^2^ = 0.0%

**Table 6:** Absolute, relative, and concentration-based wealth inequalities in overall Early Childhood Development Index status within each provincial survey.

Provincial Survey	Richest–Poorest Difference, pp	Richest/Poorest PR	SII	RII	Erreygers Index
Punjab 2017–18	26.6 (23.9–29.3)	1.57 (1.50–1.64)	0.317 (0.287–0.347)	1.71 (1.62–1.80)	0.203 (0.183–0.222)
Sindh 2018–19	34.8 (30.4–39.1)	1.96 (1.79–2.14)	0.380 (0.327–0.433)	2.24 (1.99–2.51)	0.242 (0.207–0.276)
Khyber Pakhtunkhwa 2019	18.8 (14.5–23.1)	1.42 (1.31–1.53)	0.226 (0.178–0.274)	1.51 (1.39–1.65)	0.144 (0.113–0.175)
Balochistan 2019–20	35.6 (29.9–41.3)	2.36 (2.06–2.70)	0.418 (0.349–0.486)	2.97 (2.50–3.53)	0.267 (0.221–0.314)

## Data Availability

The respondent-level MICS datasets analyzed in this study are available through UNICEF MICS following registration, approval, and acceptance of the applicable data-use terms. The datasets cannot be redistributed by the authors. Analysis code and derived nonidentifiable outputs supporting the primary analyses and selected supplementary analyses are available from the corresponding author upon reasonable request, subject to the applicable MICS data-use agreement.

## Supplementary Materials

The following supporting information can be downloaded at: https://www.mdpi.com/article/10.3390/children13101314/s1, Table S1. Complete province-specific fully adjusted models for overall Early Childhood Development Index status. Table S2. Multiplicative interaction between stunting and adult stimulation in the fully adjusted province-specific models. Table S3. Sensitivity of overall Early Childhood Development Index prevalence to the operational definition of child age. Table S4. Inverse-probability-of-complete-case weighting sensitivity for selected adjusted associations. Table S5. Wealth-related inequality in overall and domain-specific Early Childhood Development Index outcomes. Table S6. Joint maternal and paternal stimulation sensitivity analysis for overall Early Childhood Development Index status. Table S7. Sensitivity of the overall Early Childhood Development Index wealth gradient to a harmonized common-asset score. Table S8. Independent validation of reconstructed Early Childhood Development Index estimates against official provincial MICS6 reports. Table S9. Age and sex disaggregation of developmental attainment. Table S10. Adult stimulation associations across fixed-sample sequential models. Table S11. Complete false discovery rate results for 45 pooled tests. Table S12. Covariate missingness and provincial caregiver context. Table S13. Provincial comparisons standardized for age and sex. Table S14. Sensitivity to functional difficulty adjustment and ECE routing. Table S15. Sensitivity to nonresponse on individual ECDI items. Figure S1. Conceptual framework for interpretation of adjusted associations.
