# Supplementary material for: Developmental Disparities and Associated Factors Among Children Aged 36–59 Months in Four Pakistani Provinces
Source: Children (Basel). 2026 Sep 27;13(10):1314. doi: 10.3390/children13101314 (PMC13703014; doi:10.3390/children13101314)
Supplement: Supplementary file 1 [file children-13-01314-s001.zip › children-4542062-supplementary.pdf]

Supplementary Materials

Developmental Disparities and Associated Factors among Children Aged 36–59 Months in Four Pakistani Provinces

Tables S1–S15 provide detailed model results, sensitivity analyses, subgroup analyses, multiple-testing results, and validation checks. Figure S1 presents the conceptual framework. Exploratory comparisons are identified as such.

Supplementary Methods

These details support the variable definitions and statistical procedures in Sections 2.2–2.6 of the manuscript. The primary analysis preserves the original coding; the alternative definitions below are sensitivity analyses.

Eligibility and ECDI scoring

Primary eligibility required UF17=1 (completed under-five interview) and UB2=3 or 4 completed years. The alternative age analysis used calculated age CAGE=36–59 months. Original ECDI items EC6–EC15 were scored by responses confirming the specified ability or behavior. Unknown or nonresponse codes did not contribute attained items. The item-response sensitivity required valid yes/no responses to all 10 items.

Exposure coding and missing responses

Stunting required HAZFLAG=0 and a height-for-age z score (HAZ2) between −6 and 6; HAZ2 < −2 denoted stunting. Primary ECE attendance was positive for UB8=1. Other responses or skips were coded as nonattendance, except explicit nonresponse to UB6 or UB8, which was missing. The stricter routing sensitivity also excluded unresolved UB8 skips when neither UB6 nor UB7 explicitly established nonattendance.

Adult stimulation counted the six activity items in which a mother, father, or another adult participated. The indicator was positive for at least four activities; explicit activity nonresponse was missing. Maternal and paternal indicators counted activities for the respective adult. At least two affirmative plaything responses established a positive indicator even if the third response was unknown; otherwise, all three responses were required. Inadequate supervision was positive when either EC3A or EC3B indicated 1–7 days. Two zero responses established a negative indicator; unresolved combinations were missing.

Survey estimation and sensitivity analyses

Primary analyses used Python 3.13; complementary verification and selected sensitivity analyses used Python 3.12. Poisson model covariance matrices used survey-weighted score contributions summed within PSUs and centered within strata. Prevalence and subgroup analyses used Taylor linearization; zero contributions from PSUs outside a subgroup were retained for domain variance estimation. Singleton strata were centered using the overall PSU-score mean. Individual model intervals used a normal approximation.

Random-effects models used restricted maximum likelihood. Modified Hartung–Knapp–Sidik–Jonkman inference constrained the variance multiplier to $q^*=\max(1,q)$. Pooled confidence intervals used a t distribution with three degrees of freedom, and prediction intervals used two degrees of freedom. Benjamini–Hochberg correction was applied to the single 45-test family; additional standardization and sensitivity analyses were not added to this family.

IPCW used survey-weighted logistic models for complete-case inclusion based on observed developmental and demographic information with missing-indicator encodings. Stabilized inverse-probability weights were truncated at the 1st and 99th percentiles and multiplied by the child sampling weight. The province-specific stabilized-weight ranges were 0.97–1.06 in Punjab, 0.95–1.08 in Sindh, 0.95–1.16 in Khyber Pakhtunkhwa, and 0.87–1.35 in Balochistan. Results from the original IPCW analysis are reported in Table S4.

Sequential stimulation models held the primary complete-case sample fixed. Stages added age and sex; residence, education, and wealth; functional difficulty and stunting; and the remaining ECE and nurturing-care variables. Models omitting functional difficulty used the same complete cases. Age–sex standardization used the equal-province mean of the four age-group-by-sex distributions, treated as fixed for variance estimation. Six independent-survey comparisons of standardized overall ECDI received Holm adjustment.

Table S1. Complete province-specific fully adjusted models for overall Early Childhood Development Index status.

Predictor	Punjab 2017–18	Sindh 2018–19	Khyber Pakhtunkhwa 2019	Balochistan 2019–20
Maternal/caretaker education: Primary vs none/pre-primary	1.02 (0.98–1.07)	1.12 (1.04–1.22)	1.06 (0.99–1.14)	1.09 (0.96–1.24)
Maternal/caretaker education: Middle vs none/pre-primary	1.09 (1.04–1.14)	1.03 (0.92–1.16)	1.14 (1.05–1.24)	0.95 (0.80–1.13)
Maternal/caretaker education: Secondary vs none/pre-primary	1.08 (1.03–1.13)	1.15 (1.06–1.26)	1.17 (1.08–1.27)	0.82 (0.71–0.94)
Maternal/caretaker education: Higher vs none/pre-primary	1.10 (1.05–1.16)	1.08 (0.98–1.19)	1.19 (1.10–1.29)	0.79 (0.66–0.94)
Wealth: Second vs poorest	1.13 (1.07–1.19)	1.14 (1.03–1.25)	1.12 (1.04–1.20)	1.19 (1.04–1.36)
Wealth: Middle vs poorest	1.16 (1.10–1.22)	1.20 (1.08–1.33)	1.17 (1.09–1.26)	1.29 (1.10–1.51)
Wealth: Fourth vs poorest	1.18 (1.12–1.25)	1.28 (1.13–1.45)	1.14 (1.06–1.24)	1.81 (1.56–2.09)
Wealth: Richest vs poorest	1.24 (1.16–1.32)	1.40 (1.23–1.59)	1.13 (1.03–1.25)	2.30 (1.98–2.66)
Age 48–59 vs 36–47 months	1.12 (1.08–1.15)	1.16 (1.10–1.22)	1.12 (1.07–1.17)	1.08 (1.02–1.14)
Female vs male	1.06 (1.04–1.09)	1.00 (0.95–1.05)	0.98 (0.94–1.03)	1.04 (0.98–1.10)
Urban vs rural	1.01 (0.98–1.05)	1.06 (0.97–1.15)	1.08 (1.00–1.17)	0.93 (0.84–1.03)
Functional difficulty vs none	0.84 (0.78–0.91)	0.57 (0.49–0.67)	0.69 (0.60–0.79)	0.81 (0.72–0.91)
Stunted vs not stunted	0.94 (0.91–0.97)	0.87 (0.83–0.92)	0.96 (0.91–1.00)	0.87 (0.81–0.94)
Currently attending ECE vs not	1.27 (1.24–1.31)	1.32 (1.24–1.41)	1.33 (1.26–1.41)	1.11 (0.93–1.33)
Adult stimulation ≥4 vs <4 activities	1.12 (1.09–1.15)	1.08 (1.02–1.15)	1.04 (0.99–1.09)	0.92 (0.85–1.00)
≥3 children’s books vs fewer	1.13 (1.08–1.19)	1.28 (1.19–1.38)	1.11 (1.00–1.23)	1.52 (1.37–1.68)
≥2 plaything types vs fewer	0.98 (0.95–1.01)	1.14 (1.07–1.22)	1.14 (1.08–1.21)	1.59 (1.42–1.77)
Inadequate supervision vs none	1.04 (1.00–1.08)	0.97 (0.91–1.04)	1.02 (0.97–1.08)	0.98 (0.90–1.06)

Values are aPRs (95% confidence intervals) from complete-case, survey-weighted modified Poisson models. All listed predictors were entered simultaneously. Reference categories are shown after “vs”; for maternal/caretaker education the reference is none/pre-primary, and for wealth it is the poorest quintile. Province-specific model sample sizes were Punjab 15,082, Sindh 7,309, Khyber Pakhtunkhwa 9,102, and Balochistan 9,452.

Table S2. Multiplicative interaction between stunting and adult stimulation in the fully adjusted province-specific models.

**Provincial survey**	**Interaction PR (95% CI)**	**p**	**n**
Punjab 2017–18	1.062 (0.996–1.133)	0.065	15,082
Sindh 2018–19	1.027 (0.926–1.139)	0.608	7,309
Khyber Pakhtunkhwa 2019	0.992 (0.905–1.088)	0.866	9,102
Balochistan 2019–20	1.084 (0.950–1.238)	0.232	9,452

The interaction PR is the exponentiated coefficient for stunting × adult stimulation (≥4 vs <4 activities) in the fully adjusted model. A value of 1 indicates no multiplicative interaction; values >1 or <1 indicate that the joint association is respectively greater or smaller than the product of the two main effects. None of the four interaction tests met p<0.05. n is the complete-case model sample.

Table S3. Sensitivity of overall Early Childhood Development Index prevalence to the operational definition of child age.

Provincial survey	UB2 age n	Exact 36–59 n	UB2 ECDI %	Exact-age ECDI %	Difference, pp
Punjab 2017–18	15,973	15,996	**59.421**	**59.208**	**-0.213**
Sindh 2018–19	7,892	7,883	**47.450**	**47.233**	**-0.218**
Khyber Pakhtunkhwa 2019	10,102	10,046	**54.647**	**54.555**	**-0.091**
Balochistan 2019–20	12,100	12,034	**39.104**	**39.078**	**-0.026**

UB2=3 or 4 is the completed-year age definition used in official MICS6 tabulations. Exact age uses the calculated-age variable restricted to 36–59 completed months. Difference is exact-age prevalence minus UB2-based prevalence in percentage points. The small differences support the robustness of the primary age definition. Percentages and differences are displayed to three decimals; differences are calculated before rounding.

Table S4. Inverse-probability-of-complete-case weighting sensitivity for selected adjusted associations.

Provincial survey	Predictor	Complete-case aPR (95% CI)	IPCW aPR (95% CI)
Punjab 2017–18	Stunting	0.94 (0.91–0.97)	0.93 (0.90–0.97)
Punjab 2017–18	Current ECE	1.27 (1.24–1.31)	1.28 (1.24–1.32)
Punjab 2017–18	Functional difficulty	0.84 (0.78–0.91)	0.84 (0.78–0.90)
Punjab 2017–18	Adult stimulation ≥4	1.12 (1.09–1.15)	1.12 (1.09–1.16)
Punjab 2017–18	≥3 books	1.13 (1.08–1.19)	1.13 (1.08–1.19)
Punjab 2017–18	≥2 plaything types	0.98 (0.95–1.01)	0.98 (0.95–1.01)
Punjab 2017–18	Inadequate supervision	1.04 (1.00–1.08)	1.04 (1.00–1.08)
Sindh 2018–19	Stunting	0.87 (0.83–0.92)	0.87 (0.82–0.92)
Sindh 2018–19	Current ECE	1.32 (1.24–1.41)	1.33 (1.25–1.41)
Sindh 2018–19	Functional difficulty	0.57 (0.49–0.67)	0.57 (0.48–0.67)
Sindh 2018–19	Adult stimulation ≥4	1.08 (1.02–1.15)	1.09 (1.02–1.15)
Sindh 2018–19	≥3 books	1.28 (1.19–1.38)	1.29 (1.19–1.39)
Sindh 2018–19	≥2 plaything types	1.14 (1.07–1.22)	1.15 (1.07–1.23)
Sindh 2018–19	Inadequate supervision	0.97 (0.91–1.04)	0.97 (0.91–1.04)
Khyber Pakhtunkhwa 2019	Stunting	0.96 (0.91–1.00)	0.96 (0.91–1.00)
Khyber Pakhtunkhwa 2019	Current ECE	1.33 (1.26–1.41)	1.33 (1.26–1.41)
Khyber Pakhtunkhwa 2019	Functional difficulty	0.69 (0.60–0.79)	0.68 (0.60–0.78)
Khyber Pakhtunkhwa 2019	Adult stimulation ≥4	1.04 (0.99–1.09)	1.04 (0.99–1.09)
Khyber Pakhtunkhwa 2019	≥3 books	1.11 (1.00–1.23)	1.11 (1.00–1.23)
Khyber Pakhtunkhwa 2019	≥2 plaything types	1.14 (1.08–1.21)	1.14 (1.08–1.21)
Khyber Pakhtunkhwa 2019	Inadequate supervision	1.02 (0.97–1.08)	1.02 (0.97–1.08)
Balochistan 2019–20	Stunting	0.87 (0.81–0.94)	0.87 (0.81–0.95)
Balochistan 2019–20	Current ECE	1.11 (0.93–1.33)	1.11 (0.92–1.35)
Balochistan 2019–20	Functional difficulty	0.81 (0.72–0.91)	0.79 (0.70–0.89)
Balochistan 2019–20	Adult stimulation ≥4	0.92 (0.85–1.00)	0.91 (0.84–0.99)
Balochistan 2019–20	≥3 books	1.52 (1.37–1.68)	1.53 (1.38–1.70)
Balochistan 2019–20	≥2 plaything types	1.59 (1.42–1.77)	1.63 (1.45–1.83)
Balochistan 2019–20	Inadequate supervision	0.98 (0.90–1.06)	0.98 (0.90–1.06)

Complete-case estimates use the original child survey weight. IPCW estimates additionally multiply that weight by a stabilized inverse probability of complete-case inclusion, truncated at the 1st and 99th percentiles. Close agreement indicates limited sensitivity of selected associations to observed predictors of covariate completeness, but does not rule out bias from data missing not at random. IPCW, inverse-probability-of-complete-case weighting.

Table S5. Wealth-related inequality in overall and domain-specific Early Childhood Development Index outcomes.

Provincial survey	Outcome	SII (95% CI)	RII (95% CI)	Erreygers index (95% CI)
Punjab 2017–18	Overall ECDI	0.317 (0.287–0.347)	1.71 (1.62–1.80)	0.203 (0.183–0.222)
Punjab 2017–18	Literacy-numeracy	0.440 (0.413–0.466)	5.38 (4.85–5.97)	0.281 (0.263–0.299)
Punjab 2017–18	Physical	0.021 (0.011–0.031)	1.02 (1.01–1.03)	0.013 (0.007–0.020)
Punjab 2017–18	Learning	0.017 (0.002–0.032)	1.02 (1.00–1.03)	0.011 (0.002–0.020)
Punjab 2017–18	Social-emotional	0.135 (0.102–0.168)	1.30 (1.22–1.38)	0.086 (0.065–0.107)
Sindh 2018–19	Overall ECDI	0.380 (0.327–0.433)	2.24 (1.99–2.51)	0.242 (0.207–0.276)
Sindh 2018–19	Literacy-numeracy	0.496 (0.449–0.544)	9.41 (7.05–12.54)	0.316 (0.284–0.347)
Sindh 2018–19	Physical	0.067 (0.040–0.094)	1.08 (1.04–1.11)	0.043 (0.026–0.060)
Sindh 2018–19	Learning	0.180 (0.138–0.221)	1.26 (1.20–1.33)	0.114 (0.088–0.141)
Sindh 2018–19	Social-emotional	0.009 (-0.043–0.061)	1.02 (0.92–1.12)	0.006 (-0.028–0.039)
Khyber Pakhtunkhwa 2019	Overall ECDI	0.226 (0.178–0.274)	1.51 (1.39–1.65)	0.144 (0.113–0.175)
Khyber Pakhtunkhwa 2019	Literacy-numeracy	0.294 (0.254–0.334)	3.43 (2.92–4.03)	0.188 (0.161–0.215)
Khyber Pakhtunkhwa 2019	Physical	0.020 (0.000–0.040)	1.02 (1.00–1.04)	0.013 (0.000–0.026)
Khyber Pakhtunkhwa 2019	Learning	0.077 (0.035–0.119)	1.10 (1.04–1.16)	0.049 (0.023–0.076)
Khyber Pakhtunkhwa 2019	Social-emotional	0.055 (0.008–0.102)	1.10 (1.01–1.19)	0.035 (0.005–0.065)
Balochistan 2019–20	Overall ECDI	0.418 (0.349–0.486)	2.97 (2.50–3.53)	0.267 (0.221–0.314)
Balochistan 2019–20	Literacy-numeracy	0.119 (0.078–0.160)	2.27 (1.72–3.00)	0.076 (0.050–0.103)
Balochistan 2019–20	Physical	0.061 (0.035–0.087)	1.07 (1.04–1.10)	0.039 (0.022–0.056)
Balochistan 2019–20	Learning	0.265 (0.205–0.325)	1.55 (1.41–1.71)	0.170 (0.130–0.210)
Balochistan 2019–20	Social-emotional	0.264 (0.204–0.323)	1.55 (1.40–1.70)	0.169 (0.130–0.208)

SII, RII, and Erreygers indices were calculated separately for each outcome and provincial survey. SII is an absolute prevalence difference across the full fractional wealth rank and is displayed on the 0–1 prevalence-proportion scale; RII is the corresponding prevalence ratio. Positive SII and Erreygers values and RII >1 indicate pro-rich inequality. Values are descriptive and unadjusted.

Table S6. Joint maternal and paternal stimulation sensitivity analysis for overall Early Childhood Development Index status.

Survey/synthesis	Predictor	aPR (95% CI)	p or heterogeneity
Punjab 2017–18	Maternal stimulation ≥4 activities	1.11 (1.06–1.15)	<0.001
Punjab 2017–18	Paternal stimulation ≥4 activities	1.08 (1.01–1.15)	0.025
Sindh 2018–19	Maternal stimulation ≥4 activities	0.90 (0.84–0.97)	0.007
Sindh 2018–19	Paternal stimulation ≥4 activities	0.75 (0.66–0.84)	<0.001
Khyber Pakhtunkhwa 2019	Maternal stimulation ≥4 activities	0.91 (0.84–1.00)	0.039
Khyber Pakhtunkhwa 2019	Paternal stimulation ≥4 activities	1.08 (0.97–1.22)	0.162
Balochistan 2019–20	Maternal stimulation ≥4 activities	0.91 (0.83–0.99)	0.034
Balochistan 2019–20	Paternal stimulation ≥4 activities	0.85 (0.72–1.00)	0.046
Random-effects pooled	Maternal stimulation ≥4 activities	0.96 (0.81–1.13)	I^2=92.3%
Random-effects pooled	Paternal stimulation ≥4 activities	0.93 (0.69–1.25)	I^2=91.3%

Maternal and paternal stimulation indicators (≥4 of six activities in the previous 3 days) were entered jointly in place of the combined adult-stimulation indicator; all other covariates were unchanged from the primary model. Province-specific values are aPRs (95% confidence intervals). Pooled rows use random-effects synthesis; I^2 quantifies between-survey heterogeneity. These caregiver-specific estimates were exploratory and highly heterogeneous.

Table S7. Sensitivity of the overall Early Childhood Development Index wealth gradient to a harmonized common-asset score.

Provincial survey	Standard MICS gap, pp	Common-asset gap, pp	Common-asset top/bottom PR
Punjab 2017–18	26.6	20.6	1.44
Sindh 2018–19	34.8	28.8	1.76
Khyber Pakhtunkhwa 2019	18.8	16.9	1.37
Balochistan 2019–20	35.6	20.9	1.76

The standard MICS gap is the richest–poorest prevalence difference using each survey's own asset-based wealth quintiles. The common-asset analysis used an equal-weight score from 14 identically coded assets and common pooled cut points after equalizing the total survey weight contributed by each province. The sensitivity analysis tests whether pro-rich gradients persist under a more comparable asset definition; it does not create a direct income measure.

Table S8. Independent validation of reconstructed Early Childhood Development Index estimates against official provincial MICS6 reports.

Provincial survey	Indicator	Official report %	Reconstructed %	Difference, pp
Punjab 2017–18	Overall ECDI	59.4	59.42	+0.02
Punjab 2017–18	Literacy-numeracy	27.2	27.22	+0.02
Sindh 2018–19	Overall ECDI	47.5	47.45	-0.05
Sindh 2018–19	Literacy-numeracy	23.6	23.61	+0.01
Khyber Pakhtunkhwa 2019	Overall ECDI	54.6	54.65	+0.05
Khyber Pakhtunkhwa 2019	Literacy-numeracy	24.4	24.39	-0.01
Balochistan 2019–20	Overall ECDI	39.1	39.10	+0.00
Balochistan 2019–20	Literacy-numeracy	14.6	14.64	+0.04

Difference is reconstructed minus official report prevalence in percentage points. Official figures were taken from Table TC.11.1 of each provincial survey report; reconstructed estimates used the respondent-level child file, original survey design variables, and official MICS6 scoring rules. The maximum absolute discrepancy across these comparisons was approximately 0.05 percentage points, consistent with rounding of the official one-decimal tabulations.

Table S9. Age and sex disaggregation of developmental attainment

Panel A Age

Province	Group	n	Overall ECDI	Literacy–numeracy	Physical	Learning	Social–emotional
Punjab	36–47 months	8296	53.9 (52.7–55.1)	16.2 (15.3–17.1)	97.0 (96.6–97.3)	92.5 (91.9–93.1)	51.8 (50.6–53.0)
Punjab	48–59 months	7677	65.3 (64.2–66.5)	39.1 (37.8–40.3)	97.5 (97.1–97.9)	94.3 (93.8–94.9)	51.6 (50.4–52.8)
Sindh	36–47 months	3833	43.0 (41.1–44.8)	18.6 (17.1–20.0)	90.1 (88.9–91.2)	75.5 (74.0–77.1)	53.2 (51.3–55.1)
Sindh	48–59 months	4059	51.8 (49.9–53.7)	28.5 (26.7–30.2)	92.1 (91.1–93.1)	78.5 (77.0–80.0)	55.7 (53.9–57.4)
KP	36–47 months	5365	50.8 (49.0–52.6)	18.2 (16.8–19.6)	93.3 (92.6–94.1)	81.0 (79.6–82.4)	57.1 (55.5–58.8)
KP	48–59 months	4737	59.0 (57.3–60.7)	31.5 (29.7–33.2)	94.4 (93.6–95.1)	82.9 (81.5–84.3)	58.8 (57.1–60.5)
Balochistan	36–47 months	5947	37.3 (35.0–39.6)	11.6 (10.3–12.9)	90.6 (89.6–91.5)	59.3 (57.2–61.3)	60.7 (58.6–62.8)
Balochistan	48–59 months	6153	40.8 (38.5–43.2)	17.5 (15.9–19.1)	91.1 (90.1–92.1)	61.8 (59.6–64.0)	60.8 (58.7–62.9)

Panel B Sex

Province	Group	n	Overall ECDI	Literacy–numeracy	Physical	Learning	Social–emotional
Punjab	Male	8213	57.5 (56.3–58.7)	26.5 (25.4–27.5)	97.2 (96.9–97.6)	93.2 (92.6–93.8)	49.3 (48.1–50.4)
Punjab	Female	7760	61.4 (60.3–62.6)	28.0 (26.9–29.1)	97.2 (96.8–97.6)	93.6 (93.0–94.2)	54.3 (53.0–55.5)
Sindh	Male	4135	47.6 (45.6–49.5)	24.2 (22.6–25.9)	91.3 (90.2–92.3)	78.1 (76.7–79.6)	53.4 (51.6–55.2)
Sindh	Female	3757	47.3 (45.4–49.3)	22.9 (21.2–24.7)	91.0 (89.9–92.1)	75.8 (74.2–77.4)	55.6 (53.8–57.5)
KP	Male	5302	54.8 (53.1–56.5)	24.5 (22.9–26.1)	94.0 (93.3–94.7)	81.7 (80.4–83.1)	57.5 (55.9–59.2)
KP	Female	4800	54.5 (52.6–56.4)	24.2 (22.6–25.9)	93.6 (92.8–94.5)	82.1 (80.5–83.6)	58.3 (56.5–60.2)
Balochistan	Male	6252	38.2 (36.0–40.5)	14.8 (13.3–16.3)	91.1 (90.2–92.1)	60.6 (58.6–62.6)	60.2 (58.1–62.3)
Balochistan	Female	5848	40.1 (37.6–42.5)	14.5 (13.0–16.0)	90.5 (89.5–91.6)	60.5 (58.3–62.7)	61.4 (59.4–63.4)

Values are survey-weighted percentages (95% confidence intervals); n is unweighted. Age groups follow the original recorded-age definition (3 or 4 completed years). Zero-score PSUs outside each subgroup were retained in domain variance estimation. These descriptive comparisons do not estimate causal age or sex effects. KP, Khyber Pakhtunkhwa.

Table S10. Adult stimulation associations across fixed-sample sequential models

Province	n	Unadjusted	Demographic	Socioeconomic	Health	Fully adjusted
**Punjab**	**15082**	**1.288 (1.252–1.324)**	**1.292 (1.256–1.328)**	**1.140 (1.106–1.175)**	**1.136 (1.102–1.171)**	**1.119 (1.086–1.154)**
**Sindh**	**7309**	**1.235 (1.163–1.312)**	**1.232 (1.161–1.308)**	**1.127 (1.061–1.198)**	**1.123 (1.058–1.192)**	**1.085 (1.022–1.151)**
**KP**	**9102**	**1.135 (1.082–1.192)**	**1.131 (1.078–1.187)**	**1.072 (1.020–1.126)**	**1.064 (1.012–1.118)**	**1.038 (0.988–1.091)**
**Balochistan**	**9452**	**0.922 (0.838–1.014)**	**0.920 (0.836–1.011)**	**0.902 (0.826–0.986)**	**0.910 (0.836–0.990)**	**0.920 (0.850–0.995)**

Values are prevalence ratios (95% confidence intervals) for ≥4 versus <4 adult-stimulation activities. Each provincial sequence uses the primary complete-case sample throughout. Demographic adjustment adds age and sex; socioeconomic adjustment additionally adds residence, education, and wealth; health adjustment additionally adds functional difficulty and stunting; the full model additionally includes ECE, books, playthings, and supervision. Staged estimates are descriptive diagnostics, not mediation effects.

In Balochistan the point estimate is inverse at every stage. In the larger sample with observed stimulation, the unadjusted estimate is 0.931 (0.850–1.019). Neither unadjusted interval excludes 1. The direction is therefore not a reversal created solely by simultaneous adjustment.

Table S11. Complete false discovery rate results for 45 pooled tests

The Benjamini–Hochberg family comprised nine predictors crossed with five outcomes (45 tests). Pooled estimates used REML and modified Hartung–Knapp inference for four surveys. q values were calculated from the unrounded p values. Age–sex standardization and other sensitivity analyses were not included in this 45-test family.

Overall ECDI

Predictor	Pooled aPR (95% CI)	I^2 (%)	p	q
Functional difficulty vs none	0.73 (0.55–0.96)	86.5	0.036	0.1147
Stunted vs not stunted	0.92 (0.85–0.98)	63.8	0.030	0.1048
Current ECE attendance vs none	1.29 (1.22–1.37)	39.3	<0.001	0.0263
Adult stimulation ≥4 vs <4 activities	1.04 (0.91–1.19)	87.4	0.391	0.4757
≥3 children's books vs fewer	1.25 (0.99–1.57)	90.4	0.054	0.1425
≥2 plaything types vs fewer	1.19 (0.86–1.63)	96.7	0.186	0.3103
Inadequate supervision vs none	1.01 (0.96–1.07)	23.8	0.491	0.5519
Age 48–59 vs 36–47 months	1.12 (1.08–1.16)	21.3	0.002	0.0263
Female vs male	1.02 (0.96–1.09)	72.6	0.328	0.4215

Literacy–numeracy

Predictor	Pooled aPR (95% CI)	I^2 (%)	p	q
Functional difficulty vs none	0.91 (0.75–1.10)	45.1	0.210	0.3259
Stunted vs not stunted	0.79 (0.71–0.87)	31.6	0.004	0.0374
Current ECE attendance vs none	2.11 (1.66–2.69)	89.5	0.002	0.0263
Adult stimulation ≥4 vs <4 activities	1.49 (1.16–1.91)	81.0	0.014	0.0729
≥3 children's books vs fewer	1.40 (1.26–1.55)	0.0	0.002	0.0263
≥2 plaything types vs fewer	1.28 (0.91–1.80)	94.3	0.107	0.2144
Inadequate supervision vs none	1.15 (1.01–1.30)	54.3	0.040	0.1209
Age 48–59 vs 36–47 months	1.49 (1.25–1.76)	85.6	0.005	0.0374
Female vs male	1.00 (0.90–1.10)	61.9	0.883	0.9028

Physical

Predictor	Pooled aPR (95% CI)	I² (%)	p	q
**Functional difficulty vs none**	**0.95 (0.91–0.98)**	**45.1**	**0.015**	**0.0729**
**Stunted vs not stunted**	**1.00 (0.98–1.02)**	**69.2**	**0.509**	**0.5588**
**Current ECE attendance vs none**	**1.03 (1.00–1.05)**	**73.3**	**0.051**	**0.1425**
**Adult stimulation ≥4 vs <4 activities**	**1.00 (0.97–1.03)**	**80.4**	**0.919**	**0.9188**
**≥3 children's books vs fewer**	**1.02 (0.97–1.06)**	**66.5**	**0.314**	**0.4215**
**≥2 plaything types vs fewer**	**1.01 (1.00–1.02)**	**0.0**	**0.020**	**0.0903**
**Inadequate supervision vs none**	**0.99 (0.97–1.02)**	**70.1**	**0.294**	**0.4127**
**Age 48–59 vs 36–47 months**	**1.01 (0.99–1.02)**	**46.3**	**0.171**	**0.2961**
**Female vs male**	**1.00 (0.99–1.00)**	**0.0**	**0.319**	**0.4215**

Learning

Predictor	Pooled aPR (95% CI)	I^2 (%)	p	q
Functional difficulty vs none	0.84 (0.73–0.96)	84.2	0.026	0.1048
Stunted vs not stunted	0.97 (0.91–1.03)	84.6	0.162	0.2917
Current ECE attendance vs none	1.06 (0.99–1.14)	88.1	0.063	0.1576
Adult stimulation ≥4 vs <4 activities	0.96 (0.84–1.09)	94.4	0.358	0.4474
≥3 children's books vs fewer	1.08 (0.90–1.31)	95.7	0.276	0.4079
≥2 plaything types vs fewer	1.22 (0.92–1.61)	98.6	0.110	0.2144
Inadequate supervision vs none	1.01 (0.93–1.10)	89.9	0.645	0.6748
Age 48–59 vs 36–47 months	1.02 (1.00–1.03)	20.1	0.079	0.1868
Female vs male	0.99 (0.97–1.02)	38.2	0.476	0.5490

Social–emotional

Predictor	Pooled aPR (95% CI)	I² (%)	p	q
**Functional difficulty vs none**	**0.92 (0.86–0.98)**	**0.0**	**0.030**	**0.1048**
**Stunted vs not stunted**	**0.98 (0.94–1.02)**	**9.9**	**0.202**	**0.3247**
**Current ECE attendance vs none**	**0.96 (0.80–1.15)**	**68.2**	**0.533**	**0.5711**
**Adult stimulation ≥4 vs <4 activities**	**0.98 (0.92–1.05)**	**66.4**	**0.467**	**0.5490**
**≥3 children's books vs fewer**	**1.11 (0.95–1.29)**	**70.2**	**0.132**	**0.2483**
**≥2 plaything types vs fewer**	**0.88 (0.82–0.94)**	**68.1**	**0.008**	**0.0499**
**Inadequate supervision vs none**	**0.95 (0.85–1.07)**	**85.9**	**0.281**	**0.4079**
**Age 48–59 vs 36–47 months**	**1.02 (0.99–1.06)**	**0.0**	**0.093**	**0.2033**
**Female vs male**	**1.04 (0.99–1.10)**	**70.0**	**0.095**	**0.2033**

Table S12. Covariate missingness and provincial caregiver context

Panel A Missing covariates

Covariate	Punjab	Sindh	KP	Balochistan
Stunted vs not stunted	708 (4.43%)	383 (4.85%)	329 (3.26%)	859 (7.10%)
Current ECE attendance vs none	6 (0.04%)	15 (0.19%)	183 (1.81%)	42 (0.35%)
Adult stimulation ≥4 vs <4 activities	17 (0.11%)	30 (0.38%)	62 (0.61%)	236 (1.95%)
≥3 children's books vs fewer	0 (0.00%)	0 (0.00%)	11 (0.11%)	0 (0.00%)
≥2 plaything types vs fewer	20 (0.13%)	29 (0.37%)	85 (0.84%)	613 (5.07%)
Inadequate supervision vs none	151 (0.95%)	141 (1.79%)	399 (3.95%)	1,272 (10.51%)
Maternal/caretaker education	0 (0.00%)	1 (0.01%)	0 (0.00%)	2 (0.02%)

Counts and percentages are unweighted among eligible children. Exclusions overlap and must not be summed. Age, sex, residence, functional difficulty, and wealth were complete in the primary samples.

Panel B Child population and caregiver characteristics

Characteristic	Punjab	Sindh	KP	Balochistan
Urban residence	34.2	44.7	14.9	26.1
No/pre-primary caregiver education	45.1	67.0	69.6	82.9
Primary caregiver education	19.5	11.1	10.6	5.2
Middle caregiver education	10.2	5.1	5.7	2.5
Secondary caregiver education	12.8	7.8	6.8	6.6
Higher caregiver education	12.5	9.0	7.3	2.7

Values are survey-weighted percentages of eligible children with observed data. These describe the caregivers and residence of the surveyed child population, not province-wide adult literacy or urbanization rates.

Table S13. Provincial comparisons standardized for age and sex

Province	Overall ECDI	Literacy–numeracy	Physical	Learning	Social–emotional
Punjab	59.5 (58.6–60.4)	27.4 (26.6–28.2)	97.2 (97.0–97.5)	93.4 (93.0–93.8)	51.7 (50.8–52.6)
Sindh	47.3 (45.8–48.8)	23.4 (22.1–24.8)	91.1 (90.2–91.9)	77.0 (75.8–78.1)	54.4 (53.0–55.8)
KP	54.9 (53.5–56.2)	24.7 (23.4–26.0)	93.8 (93.3–94.4)	81.9 (80.8–83.1)	58.0 (56.7–59.3)
Balochistan	39.0 (37.0–41.1)	14.5 (13.3–15.8)	90.8 (90.1–91.6)	60.5 (58.7–62.3)	60.8 (59.0–62.5)

Direct standardization uses the mean of the four survey-weighted provincial distributions across age group × sex cells, giving each province equal contribution. Fixed reference proportions in order younger male, younger female, older male, older female are 0.264185, 0.243307, 0.256437, 0.236070. These are comparative reference weights, not national population weights. Intervals condition on this fixed reference and retain each survey's strata and PSUs.

Pairwise differences in standardized overall ECDI

Comparison	Difference in percentage points (95% CI)	Holm-adjusted p
Punjab minus Sindh	12.21 (10.46–13.97)	<0.001
Punjab minus KP	4.65 (3.04–6.26)	<0.001
Punjab minus Balochistan	20.47 (18.25–22.69)	<0.001
Sindh minus KP	-7.56 (-9.61–-5.52)	<0.001
Sindh minus Balochistan	8.26 (5.70–10.81)	<0.001
KP minus Balochistan	15.82 (13.36–18.28)	<0.001

Intervals use independent-survey variances. Holm correction covers the six overall ECDI comparisons. These exploratory contrasts remain confounded by survey period and other provincial differences; they are not causal province effects.

Table S14. Sensitivity to functional difficulty adjustment and ECE routing

Panel A Omit functional difficulty on the same complete-case sample

Province	n	ECE attendance	Stunting	Adult stimulation
Punjab	15082	1.28 (1.24–1.32)	0.93 (0.90–0.96)	1.12 (1.09–1.16)
Sindh	7309	1.33 (1.25–1.41)	0.87 (0.82–0.92)	1.08 (1.02–1.15)
KP	9102	1.34 (1.27–1.42)	0.95 (0.91–1.00)	1.05 (0.99–1.10)
Balochistan	9452	1.10 (0.92–1.32)	0.87 (0.81–0.94)	0.92 (0.85–1.00)

Panel B Apply stricter ECE skip classification

Province	n	ECE attendance	Stunting	Adult stimulation
Punjab	15082	1.27 (1.24–1.31)	0.94 (0.91–0.97)	1.12 (1.09–1.15)
Sindh	7309	1.32 (1.24–1.41)	0.87 (0.83–0.92)	1.08 (1.02–1.15)
KP	9102	1.33 (1.26–1.41)	0.96 (0.91–1.00)	1.04 (0.99–1.09)
Balochistan	9451	1.11 (0.93–1.33)	0.87 (0.81–0.94)	0.92 (0.85–1.00)

Values are aPRs (95% confidence intervals) for overall ECDI. Panel A removes only functional difficulty from the primary model, retaining its original complete cases. This does not resolve shared reporting or construct overlap. Panel B additionally codes an unresolved current-attendance skip as missing unless an explicit UB6 or UB7 response establishes nonattendance; direct UB8 responses and primary nonresponse exclusions are retained. The stricter rule removes one additional complete case in Balochistan and none in the other provinces.

Table S15. Sensitivity to nonresponse on individual ECDI items

Province	Any unknown/nonresponse n	All items valid n	Primary overall ECDI	All items valid overall ECDI
**Punjab**	**403**	**15570**	**59.4 (58.6–60.3)**	**59.8 (58.9–60.7)**
**Sindh**	**359**	**7533**	**47.5 (45.9–49.0)**	**48.2 (46.6–49.7)**
**KP**	**719**	**9383**	**54.6 (53.3–56.0)**	**55.1 (53.7–56.5)**
**Balochistan**	**2229**	**9871**	**39.1 (37.1–41.2)**	**42.6 (40.3–44.8)**

Primary ECDI scoring counts only the response confirming the specified ability or behavior toward item attainment. The sensitivity analysis includes only children with valid yes/no responses on all EC6–EC15 items and retains domain survey variances. Percentages (95% confidence intervals) are survey-weighted; n is unweighted. The restriction changes the sample and cannot determine which estimate is unbiased. Differences are largest in Balochistan, so item nonresponse remains a limitation despite preserved province ordering.

Figure S1. Conceptual framework for interpretation of adjusted associations

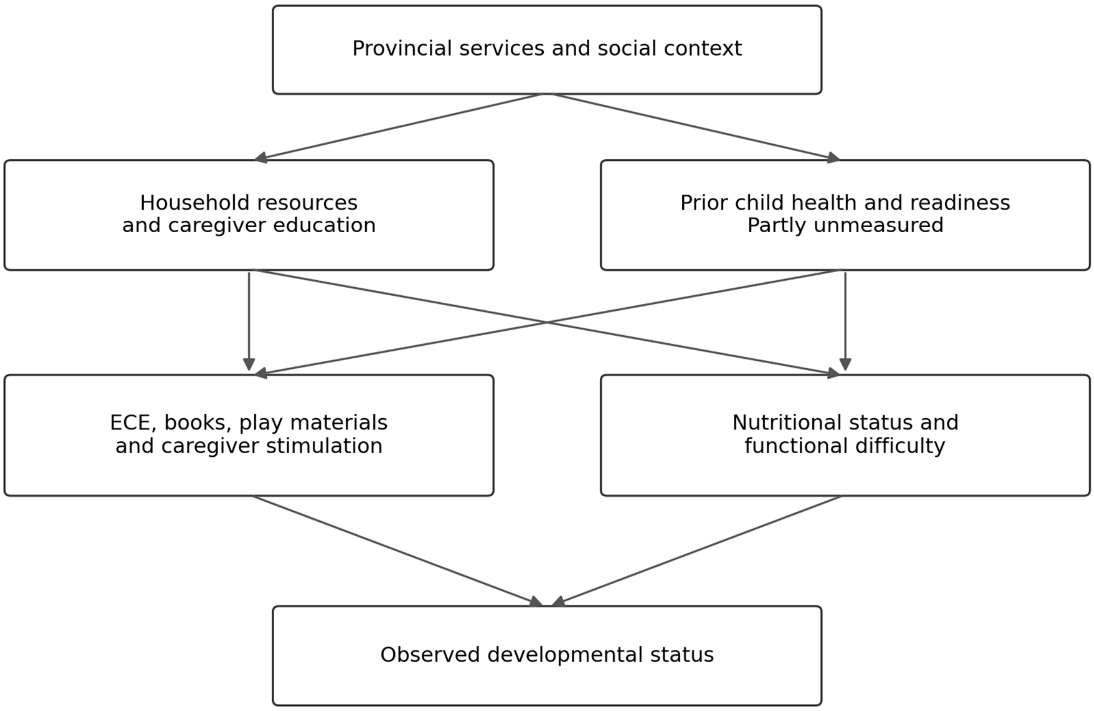

Arrows represent hypothesized pathways, not relationships established by these data. Prior developmental readiness may influence both ECE enrollment and caregiver responses to developmental concerns. Household resources may influence development through several proximal pathways. Age and sex may influence multiple child-level nodes. Caregiver interpretation and reporting may jointly affect observed stimulation, functional difficulty, and ECDI; this shared measurement process is not depicted as a biological pathway. Survey period is inseparable from provincial context. The figure explains why mutually adjusted coefficients are conditional associations and not separate total causal effects.
